# Simultaneous determination of hydrophilic and lipophilic constituents in herbal medicines using directly-coupled reversed-phase and hydrophilic interaction liquid chromatography-tandem mass spectrometry

**DOI:** 10.1038/s41598-017-07087-x

**Published:** 2017-08-01

**Authors:** Wan-Yang Sun, Qin-Wei Lu, Hao Gao, Ling Tong, Dong-Xiang Li, Zheng-Qun Zhou, Zheng-Jin Jiang, Henry Sun, Kai-Shun Bi

**Affiliations:** 1 Institute of Traditional Chinese Medicine & Natural Products, College of Pharmacy, Jinan University, Guangzhou, Guangdong 510632 China; 2 National and Local Joint Engineering Laboratory for Key Technology of Chinese Material Medica Quality Control, School of Pharmacy, Shenyang Pharmaceutical University, Shenyang, 110016 China; 3 School of Pharmacy, China Pharmaceutical University, Nanjing, 210009 China; 4 State Key Laboratory of Core Technology in Innovative Chinese Medicine, Pharmaceutical Analysis Institute, Tasly Academy, Tianjin, 300402 China; 5 Department of Pharmacy and Guangdong Province Key Laboratory of Pharmacodynamic Constituents of Traditional Chinese Medicine & New Drug Research, Jinan University, Guangzhou, 510632 China

## Abstract

Limitations in the separation ability of conventional liquid chromatography system remains a challenge in developing a versatile method for simultaneously determining both hydrophilic and lipophilic constituents in herbal medicines (HMs). To measure compounds covering a broad polarity span in HMs, we developed a directly-coupled reversed-phase and hydrophilic interaction liquid chromatography-tandem mass spectrometry system. Samples were firstly separated according to lipophilicity by using a C_18_ column. Utilizing a T-piece as connector, the eluent was then pumped into an amide column to get further separation that mainly based on the hydrogen bonding effects. Dan-Qi pair, an extensively used herb-combined prescription in China, was selected to test the practicability and performance of the established system. A total of 27 components, containing 9 hydrophilic and 18 lipophilic constituents, were simultaneously determined using a schedule multiple reaction monitoring method in 15 min. Up to 69.9% content could be monitored in one injection in Dan-Qi pair extract, showing a significant advantage over previous methods. The proposed method was expected to benefit the controllability of herbal medicines.

## Introduction

Herbal medicines (HMs) are usually derived from one or several crude materials and thereby contain numerous hydrophilic and lipophilic constituents^1–4^. They are quite different from chemical drugs in that the identities of the active constituents of HMs are not always clear^5^. Therefore, increasing the proportion of quantified constituents is of great significance to elucidate the mass balance and guarantee the quality of HMs^6, 7^. Owing to the large differences in polarities of constituents in HMs and limitation of analytical techniques, only constituents with similar polarities can be determined simultaneously. For instance, amino acids are generally determined using liquid chromatography (LC) or gas chromatography after derivatization^8, 9^, carbohydrates are always separated using hydrophilic interaction chromatography (HILIC) and detected by evaporative light scattering detector or mass spectrometry (MS)^10^, and a variety of lipophilic ingredients are usually determined using high performance liquid chromatography and LC-MS^11, 12^. As a consequence, multiple analytical methods have to be developed and implemented routinely. This makes the quantification work very tedious, and significantly increases the uncertainty and variability of determined results. Therefore, there is an urgent need to develop a rapid and sensitive method to simultaneously determine hydrophilic and lipophilic constituents.

Liquid chromatography coupled with tandem mass spectrometry (MS/MS) has been established as an important tool for the quantitative analysis of multiple non-volatile molecules in HMs^13^. Multiple reaction monitoring using characteristic precursor and product ions of each analyte can provide outstanding selectivity and sensitivity^14, 15^. The matrix effect, which is regarded as a signal enhancement or suppression of the analyte due to the co-elution of matrix components, often interferes with the performance of this method^16^. When analyzing hydrophilic and lipophilic constituents simultaneously utilizing a conventional single column, it is difficult to gain enough separation for both to reduce the influence of co-eluted analytes and matrixes. Therefore, an improvement that in terms in separating constituents across a great polarity span is needed.

The combination of reversed-phase (RP) and hydrophilic interaction liquid chromatography (HILIC) has been proven to be a powerful technique to overcome this problem. Three types of systems, including the fraction-based assembly^17, 18^, directly-coupled^19, 20^, and valve switching-based two-dimensional LC systems^21, 22^, are widely used in analyzing natural products, metabolites, and metabolomics. The off-line RP × HILIC system (fraction-based) offers the advantage of outstanding peak capacity and resolution, but it is labor-intensive and time-costing^23^. Also fractions may suffer from risks such as contamination and degradation during transfer^24^. The directly-coupled RP-HILIC combination and valve switching-based assembly are both on-line systems. The former one is typically connected using a T-piece, which can bring in another mobile phase corresponding to the subsequent separation. This type of system is simple to set up and implement, but sacrifices some resolution and sensitivity compared with each single mode^25^. The latter one, especially the comprehensive two-dimensional RP × HILIC LC system, can provide good resolution in a relatively short time. However, the employment of valve makes it difficult to establish separation systems, optimize analytical methods, and maintain stable performances^26^. Owing to the approachable construction of devices and good repeatability attributing to the online system, direct-coupled RP-HILIC system is potential to be a powerful tool in quantitative analysis of samples with complex components, *e*.*g*., human urine^19^, toxins in marine harmful algae^20^, phospholipids^27^, and *Cistanche tubulosa* ^28^.

Dan-Qi pair (DQP), a herb-combined prescription that derived from *Radix Salvia miltiorrhiza* (RSM, Danshen in Chinese) and *Radix Panax notoginseng* (RPN, Sanqi in Chinese), has been extensively used for the treatment of cardiovascular and cerebrovascular diseases in China for over 30 years^29, 30^. Up to now, at least 8 preparations of this prescription are officially registered in Chinese Pharmacopoeia^31, 32^, and one of them (Cardiotonic^®^ Pills) was approved by the US Federal Drug Administration (FDA) for Phase III clinical trials to treat chronic stable angina in 2012^33^. During the production process, DQP is generally extracted using water and/or ethanol, and consequently contains various lipophilic and hydrophilic constituents. In previous studies, the three major chemical families, including diterpenoid quinones (from RSM), phenolic acids (from RSM), and saponins (from RPN) in DQP extracts were quantified using HPLC-MS methods, but their contents were no more than 16%^34^. Our preliminary study (data not shown) also found a great amount (30–50%) of hydrophilic constituents in this prescription, such as amino acids, carbohydrates, and organic acids. Monitoring both lipophilic and hydrophilic ingredients is thus critical for clarifying the mass balance as well as the quality of DQP.

**Figure 1:**
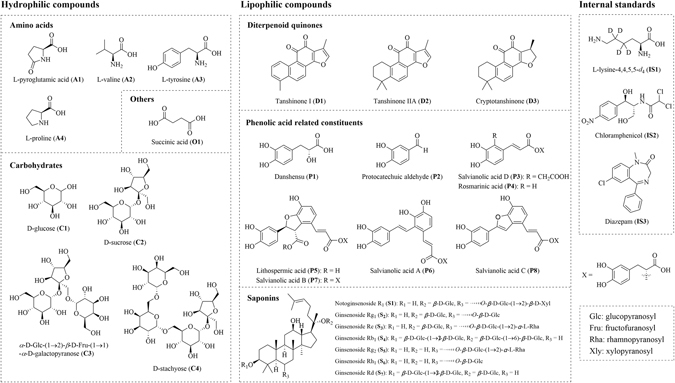
Structures of 27 analytes and 3 internal standards.

In the present work, a directly-coupled reversed-phase and hydrophilic interaction liquid chromatography-tandem mass spectrometry system (RP-HILIC-MS/MS) method was developed to simultaneously determine the content of 27 constituents in DQP extracts within 15 min. The 27 chemical markers covered most chemical families in DQP, *i*.*e*., amino acid, carbohydrate, diterpenoid, phenolic acid, and saponin. Structures of all the analytes and internal standards are presented in Fig. 1. The method was fully validated, and was used to analyze DQP extracts, RSM, and RPN samples. The results obtained from RP-HILIC-MS/MS method were further evaluated by comparing with those from conventional single column methods, namely RPLC-MS/MS and HILIC-MS/MS.

## Results and Discussion

### Selection of the extraction method

The extraction method of analytes was primarily investigated to make sure that both hydrophilic and lipophilic constituents could be extracted sufficiently. Ultrasonification in a methanol-water system showed adequate extraction efficiency for all the analytes in previous studies^14, 35^. Therefore, the optimization of the extraction method focused on ratios of organic solvent (100%, 70%, 50%, 30%, and 0%). To eliminate the content variance of each analyte, the extraction efficiency was assessed by dividing the peak area of each analyte by the maximum value of the peak areas obtained from different extraction solvents. The extraction efficiency results are shown in Fig. S1. Amino acids, carbohydrates, and succinic acid were easily extracted by water, whereas diterpenoid quinones could be extracted thoroughly in methanol, and most phenolic acids and saponins could be extracted both in water and methanol. When methanol-water solvent (70:30) was employed, the extraction efficiencies of all the analytes were not less than 85.5% with an average of 93.4%. Therefore, the ratio of methanol and water was set at 70:30.

### Optimization of the mass parameters

The MRM ion pairs for each analyte were investigated in order to obtain a sensitive and accurate quantitative response. 200–400 ng/mL of each standard solution was directly infused into the mass spectrometer with a flow rate of 20 μL/min. DP and EP were firstly optimized to gain MS spectra. Positive quasi-molecular ions ([M + H]^+^) generated the highest responses for most of the amino acids and diterpenoid quinones, except adenine and pyroglutamic acid. All the carbohydrates, phenolic acids, and saponins combined with adenine, pyroglutamic acid and succinic acid showed stronger quasi-molecular ion ([M-H]^−^) in negative mode. Next the product ion and its corresponding CE and CXP were optimized in the MRM mode. Following the legislation that more than two product ions should be selected to confirm the precursor ions^36^, the most abundant and specific ion transitions of compounds were selected as the quantitative ion pairs, and the second ones as the qualitative ion pairs (Table 1). Schedule MRM method was used to monitor the analytes only in its expected retention time window, and thus allowed increasing the sensitivity, accuracy, and reproducibility by optimizing both the cycle time and the dwell time.

**Table 1:** Retention times and optimized mass spectrometry parameters of analytes and internal standards.

NO.	Analyte	RT (min)	MS (*m*/*z*)	Precusor ion → product ion	DP (V)	EP (V)	CE (V)	CXP (V)
**Hydrophilic constituents**								
**Amino acids**								
A1	L-pyroglutamic Acid	2.08	128.1 [M-H]^−^	128.1 → 82.3^a^	−52	−10	−21	−9
				→ 71.9^b^			−26	−9
A2	L-valine	2.41	118.0 [M+H]^+^	118.0 → 72	109	8	16	12
				→ 55.0			28	6
A3	L-tyrosine	2.44	182.1 [M+H]^+^	182.1 → 136.1	79	7	18	12
				→ 165.1			16	13
A4	L-proline	2.69	115.9 [M+H]^+^	115.9 → 69.9	140	13	21	25
				→ —			—	—
**Carbohydrates**								
C1	D-glucose	3.10	179.0 [M-H]^−^	179.0 → 89.0	−124	−9	−12	−9
				→ 119.1			−12	−10
C2	D-sucrose	3.75	341.1 [M-H]^−^	341.1 → 89.2	−135	−11	−23	−6
				→ 119.4			−23	−7
C3	*α*-D-glucopyranosyl-(1→2)-*β*-D-fructofuranosyl-(1→1)-*α*-D-galactopyranose	5.16	503.2 [M-H]^−^	503.2 → 221.2	−204	−5	−37	−12
				→ 178.8			−30	−15
C4	D-stachyose	6.74	665.2 [M-H]^−^	665.2 → 383.1	−204	−5	−30	−15
				→ 179.0			−44	−13
**Others**								
O1	Succinic Acid	2.06	117.0 [M-H]^−^	117.0 → 73.1	−80	−2	−16	−16
				→ 98.9			−14	−16
**Lipophilic constituents**								
**Diterpenoid quinones**								
D1	Cryptotanshinone	11.97	297.2 [M+H]^+^	297.2 → 254.0	196	14	35	20
				→ 279.2			27	10
D2	Tanshinone I	12.13	277.1 [M+H]^+^	277.1 → 249.0	221	14	28	25
				→ 193.0			37	25
D3	Tanshinone II_A_	13.7	295.2 [M+H]^+^	295.2 → 277.2	235	11	28	10
				→ 249.2			32	8
**Phenolic acid related constituents**								
P1	Danshensu	2.22	196.7 [M-H]^−^	196.7 → 135.0	−90	−10	−24	−15
				→ 178.9			−14	−10
P2	Protocatechuic Aldehyde	3.03	137.0 [M-H]^−^	137.0 → 108.1	−95	−11	−30	−10
				→ —			—	—
P3	Salvianolic Acid D	4.61	417.1 [M-H]^−^	417.1 → 175.0	−33	−3	−27	−20
				→ 373.1			−20	−32
P4	Rosmarinic Acid	4.99	359.0 [M-H]^−^	359.0 → 161.0	−100	−12	−23	−14
				→ 196.7			−20	−12
P5	Lithospermic Acid	5.19	537.0 [M-H]^−^	537.0 → 493.1	−90	−11	−14	−16
				→ 295.0			−25	−9
P6	Salvianolic Acid A	5.59	493.0 [M-H]^−^	493.0 → 295.0	−118	−10	−23	−9
				→ 185.0			−32	−16
P7	Salvianolic Acid B	5.73	717.0 [M-H]^−^	717.0 → 519.0	−40	−10	−27	−16
				→ 321.0			−42	−30
P8	Salvianolic Acid C	6.24	491.0 [M-H]^−^	491.0 → 293.0	−118	−10	−23	−9
				→ 265.0			−29	−13
**Saponins**								
S1	Notoginsenoside R_1_	4.72	931.5 [M-H]^−^	931.5 → 637.5	−260	−9	−53	−11
				→ 799.5			−45	−13
S2	Ginsenoside Rg_1_	4.77	799.5 [M-H]^−^	799.5 → 637.5	−260	−12	−35	−11
				→ 475.6			−51	−8
S3	Ginsenoside Re	4.82	945.6 [M-H]^−^	945.6 → 637.6	−240	−9	−54	−19
				→ 783.4			−52	−19
S4	Ginsenoside Rb_1_	5.97	1107.7 [M-H]^−^	1107.7 → 323.4	−290	−10	−63	−9
				→ 945.5			−63	−16
S5	Ginsenoside Rg_2_	6.27	783.5 [M-H]^−^	783.5 → 475.4	−29	−7	−52	−16
				→ 637.4			−40	−11
S6	Ginsenoside Rh_1_	6.36	637.5 [M-H]^−^	637.5 → 161.1	−145	−12	−30	−14
				→ 475.0			−35	−14
S7	Ginsenoside Rd	6.51	945.5 [M-H]^−^	945.5 → 621.6	−190	−9	−53	−11
				→ 783.7			−50	−11
**Internal standards**								
IS1*	L-lysine−4,4,5,5-*d*_4_	4.38	151.2 [M+H]^+^	151.2 → 88.1	25	7	21	11
IS2*	Chloramphenicol	5.55	321.1 [M-H]^−^	321.1 → 152.0	−125	−10	−23	−16
IS3*	Diazepam	8.41	285.1 [M+H]^+^	285.1 → 154.2	152	7	36	20

*Internal standard, ^a^quantitative ion pair, ^b^qualitative ion pair. RT, retention time; DP, declustering potential; EP, entrance potential; CE, collision energy; CXP, cell exit potential.

### Development of the directly-coupled RP-HILIC-MS/MS method

**Figure 2:**
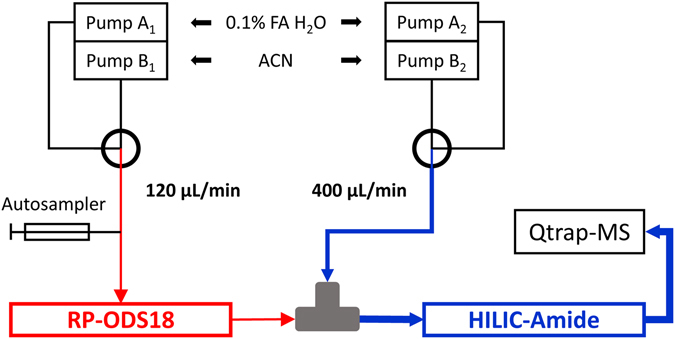
Schematic of the directly-coupled RP-HILIC-MS/MS system.

Considering the efficiency and practicality of the method, on-line combinative chromatographic systems are preferred. RPLC and HILIC can be assembled in-series or in-parallel through various kinds of interfaces, mainly splitter and value switching coupled with a trapping loop or a trapping column. Among them, directly connecting using a T-piece is a simple and practical way to establish an RP-HILIC system. This type of combination can easily avoid redundant information acquired using an in-parallel system, but will suffer from the influence of the incompatibility of mobile phase between RPLC and HILIC. To overcome this issue, another mobile phase was proposed for infusion at a high flow rate to dilute the anterior mobile phase. The schematic of the directly-coupled RP-HILIC-MS/MS system is illustrated in Fig. 2.

#### Selection of the RP and HILIC columns

The orthogonality between two separation modes are necessary in a directly-coupled system to get a complementary selectivity. In this study, RP columns containing Shim-Pack XR-ODS III (1.6 μm particles, 2.0 mm i.d. × 50 mm) and Acquity BEH C_18_ (1.7 μm particles, 2.1 mm i.d. × 50 mm), and HILIC columns containing Acquity BEH HILIC (1.7 μm particles, 2.1 mm i.d. × 100 mm) and Acquity BEH Amide (1.7 μm particles, 2.1 mm i.d. × 100 mm) were tested separately. All the RP columns showed good peak shape and selectivity for diterpenoid quinones, phenolic acids, and saponins. The XR-ODS column gave sharper peaks and more rapid separation owing to its smaller particle size and inner diameter. Almost all the hydrophilic constituents co-eluted with solvent at the dead time in RP mode. In HILIC mode, the silica column (BEH HILIC) showed poor retention of all the analytes except carbohydrates and amino acids. In contrast, amide bonded particles provided strong retention of carbohydrates and saponins, moderate retention of amino acids, and almost no retention of diterpenoid quinones and phenolic acids. Owing to the good orthogonality and separation performance, the XR-ODS and BEH Amide columns were used for further study.

#### Combination of the RP and HILIC columns

**Figure 3:**
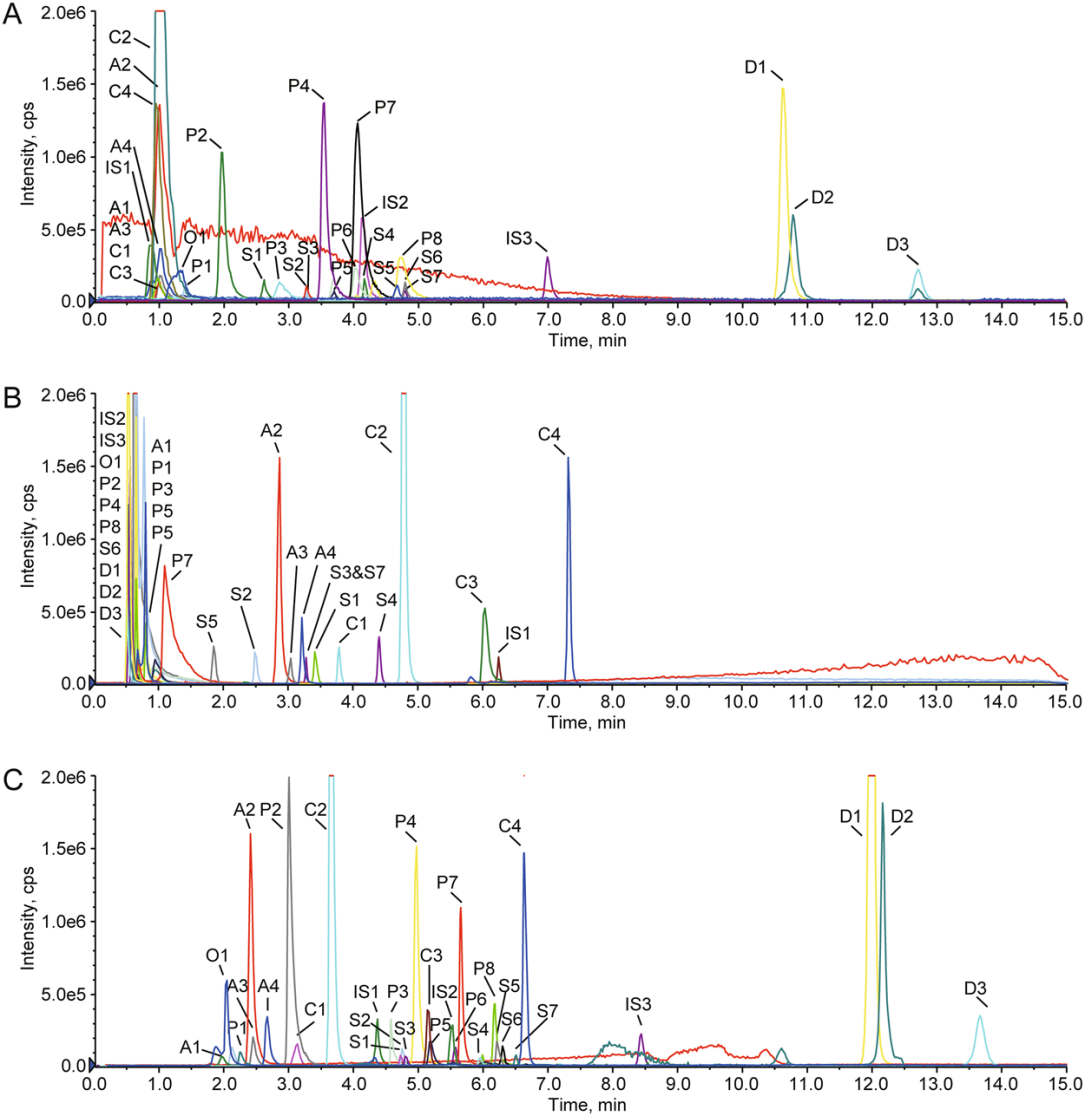
Representative total ion current (TIC) chromatograms in MRM mode for Dan-Qi pair extract and three internal standards using RPLC-MS/MS analysis (XR-ODS column) (**A**), HILIC-MS/MS analysis (BEH Amide column) (**B**), and RP-HILIC-MS/MS analysis (**C**), respectively. The analyte abbreviations are given in Table 1.

The performance of the method is highly related to the combination order of the two columns. The directly-coupled system could be established in two ways, namely HILIC-RP-MS/MS and RP-HILIC-MS/MS system. A previous study showed that HILIC mode can provide low back pressure and lead to a significant sensitivity improvement in electrospray ionization^37^. This is primarily attributed to the utilization of a highly volatile organic mobile phase, which offers weak viscosity and enhances the droplet formation and desolvation efficiency^38^. Theoretically, HILIC mode was more suitable for the secondary separation. We still performed both systems to ascertain the optimal set for separating various hydrophilic and lipophilic analytes in samples. The RP-HILIC-MS/MS system was selected for the following three reasons: (1) it provided better separation and sharper peaks, especially for amino acids and phenolic acids; (2) it gave a 2 to 3 fold enhancement in ion intensities of most analytes, which is consistent with a previous study^39^; (3) it offered a *ca*. 3000 psi reduction in system pressure. Consequently, the XR-ODS and BEH Amide columns were combined in series to establish the RP-HILIC-MS/MS system. Representative chromatograms are shown in Fig. 3.

#### Optimization of the mobile phase

**Figure 4:**
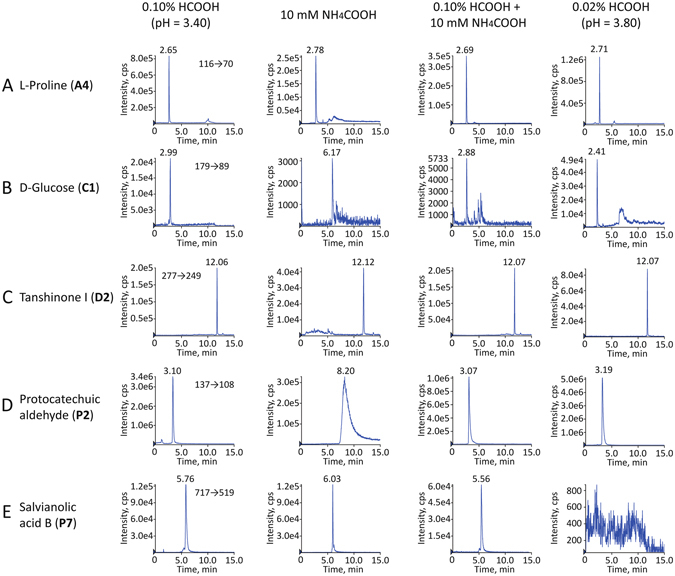
MRM chromatograms of L-proline (**A**), D-glucose (**B**), tanshinone I (**C**), protocatechuic aldehyde (**D**), and salvianolic acid B (**E**) using directly-coupled RP-HILIC-MS/MS analysis with different acidic additives (0.1% HCOOC, 10 mM NH_4_COOH, 0.1% HCOOH combined with 10 mM NH_4_COOH, and 0.02% HCOOH) in the mobile phases.

Compared with methanol, acetonitrile provided better separation, especially in HILIC mode and thereby was selected as the organic mobile phase. The selection of additives is also of vital importance to obtain good separation efficiency, peak resolution, and peak symmetry. Three additives that were used in the aqueous phase were investigated as shown in Fig. 4, including 0.10% HCOOH, 10 mM NH_4_COOH, and 0.10% HCOOH combined with 10 mM NH_4_COOH. Compared with the other two additives, formic acid significantly enhanced the responses of carbohydrates and diterpenoid quinones (e.g. D-glucose and tanshinone I as shown in Fig. 4B and C) and improved the peak shapes of pH sensitive constituents including amino acids and phenolic acids (e.g. L-proline and protocatechuic aldehyde as shown in Fig. 4A and D) owing to suppression of the ionization of acidic compounds in the mobile phase. In contrast, ammonia formate decreased the responses of almost all the analytes, and seriously tailed the peaks of phenolic acids (e.g. protocatechuic aldehyde as shown in Fig. 4D). The mixed additive could improve the shapes of peaks, but would still suffer from a certain degree of loss of sensitivity. All the additives showed almost no influence on saponins and diterpenoid quinones. Generally, the addition of formic acid will suppress the ionization of electrospray sources in the negative mode^40^. However, the results showed that most analytes exhibited higher responses than those in ammonia formate system. Of the above, formic acid was selected to further ascertain of its optimal addition level. Formic acid slightly reduced the responses of some analytes in negative polarization mode, such as carbohydrates and saponins, but significantly improved the peak shapes as well as the responses of all the phenolic acids. To minimize the suppression of other analytes monitored in negative mode, the addition level of formic acid in aqueous phase (pH 3.80, 3.60, 3.40, 3.25) was carefully investigated. We found that some phenolic acids, such as salvianolic acid B (Fig. 4E) and salvianolic acid D, were hardly detected with a pH level bigger than 3.40 in aqueous phase. Considering both separation performance and sensitivity, 0.10% formic acid (v/v, corresponding to pH 3.40) was chosen as the additive.

#### Optimization of the flow rate and elution program

The elution strengths of the same mobile phase used in RP and HILIC modes are almost opposite. In order to overcome the incompatibility of the mobile phase, we employed a high-aqueous phase in PR separation at a low flow rate and a high-acetonitrile phase in HILIC separation at a high flow rate. Because the pressure of a directly-coupled system is the accumulation of that of RP and HILIC, the total flow rate was set at less than 0.70 mL/min. With fixed 0.50 mL/min in HILIC mode, the flow rate in RP mode was first studied using five levels as follows: 0.06, 0.08, 0.10, 0.12, and 0.14 mL/min. The signal intensity increased along with a greater of flow rate, and reached a plateau at the flow rate of 0.12 mL/min. The flow rate of the HILIC mode was then investigated at 0.30, 0.40, and 0.50 mL/min, respectively, with a corresponding flow rate of 0.12 mL/min in RP mode. Satisfactory separation performances were obtained at all flow rates with a slight difference in the responses of ion pairs. Consequently, 0.40 mL/min was adopted because of the high level of responses and flexible system pressure.

The elution programs played key roles in developing the proposed method. Poor separation performances (e.g. peak broadening and peak tailing) and long retention times were observed on account of the low flow rate used in RP mode. As a consequence, a relatively high ratio of organic phase was adopted in the elution program (starting at 25% acetonitrile) to achieve a 15 min separation of all the analytes. HILIC was employed to further separate the eluent mainly based on hydrogen bonding effects. We found that diterpenoid quinones barely eluted in the HILIC column when using a conventional elution program (50% acetonitrile-water between 10.0–15.0 min). This is mainly caused by the dual HILIC-RP mechanism on HILIC columns in aqueous-organic mobile phases owing to the existence of some non-polar structural elements on the surface of the silica support^41^. A previous study has shown that in 50% acetonitrile both HILIC and RP mechanisms affect the retention simultaneously^42^, agreeing well with the retention behaviors of diterpenoid quinones in HILIC mode. Therefore, the organic phase ratio in the HILIC system was rapidly increased to 97% at 10.5 min to guarantee the direct elution of diterpenoid quinones.

### Validation of the analytical method

**Figure 5:**
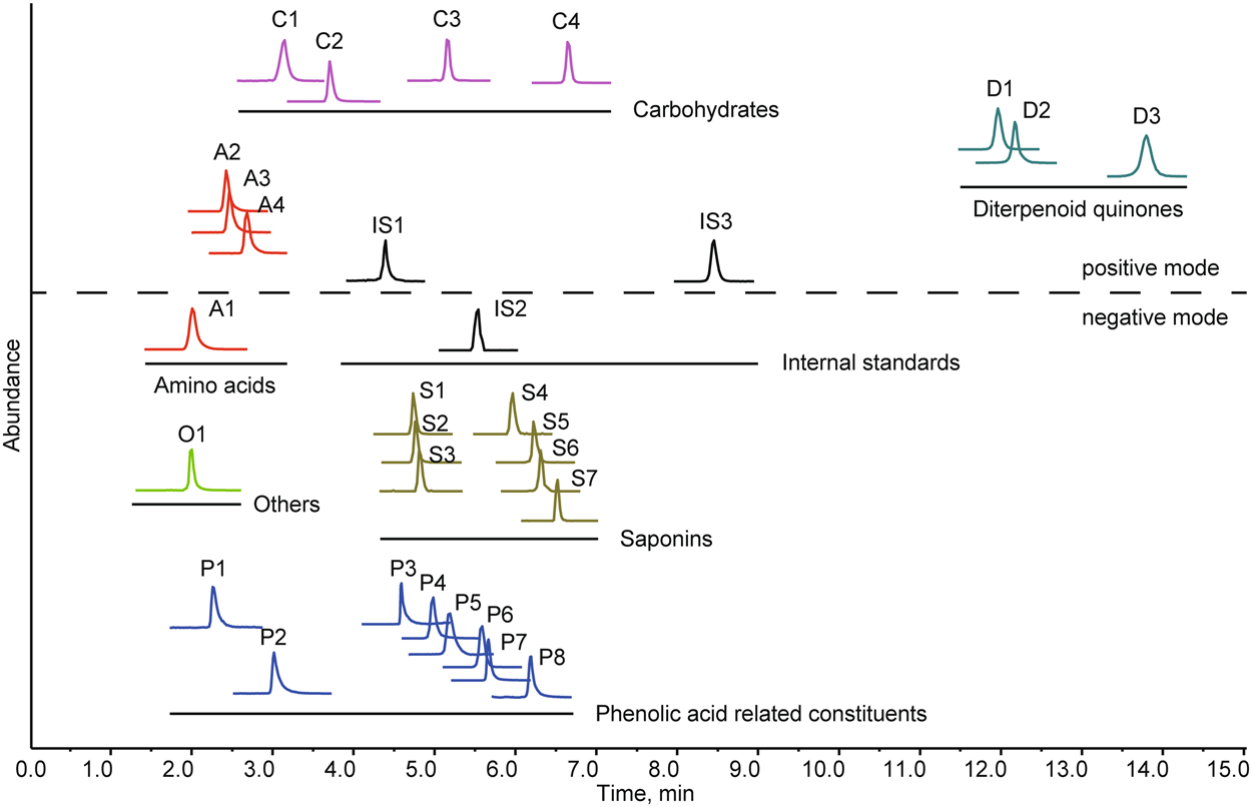
The MRM chromatograms of 27 analytes in Dan-Qi pair extract and three internal standards.

The developed directly-coupled RP-HILIC-MS/MS system was validated based on USP 36 〈1225 VALIDATION OF COMPENDIAL PROCEDURES〉 by using the internal standard method. L-lysine-4,4,5,5-*d*_4_, diazepam and chloramphenicol were employed as internal standards to test three representative samples, namely DQP extract (DQP01), RSM (RSM07), and RPN (RPN06). The results are described in the Supplemental Information. The specificity was satisfactory for all analytes owing to the high selectivity of schedule MRM as shown in Fig. 5. Table S1 lists internal standards, linear regression data, limits of detection, and limits of quantitation of 27 analytes. All analytes showed good linearity (*R* ^2^ > 0.9936) in a wide concentration range, which was benefited by the excellent dynamic range (10^4^–10^5^) of the QTrap 5500. Results for accuracy, precision, repeatability, and stability are listed in Tables S2 and S3. The overall recoveries of DQP, RSM, and RPN were 95.1–106.8% (RSD < 5.0%), 95.6–106.4% (RSD < 5.3%), and 96.0–104.5% (RSD < 4.6%) at spiked 50%, 100%, and 200%, respectively. The RSDs of the intra- and inter-day precision were 0.6–4.9% and 1.3–5.4%, respectively. The RSDs of the repeatability test were 1.1–5.6%. And the overall stability variations in 24 h were no more than 4.9%. Therefore, the proposed method is sensitive, precise, accurate, and reproducible.

### Sample analysis

**Figure 6:**
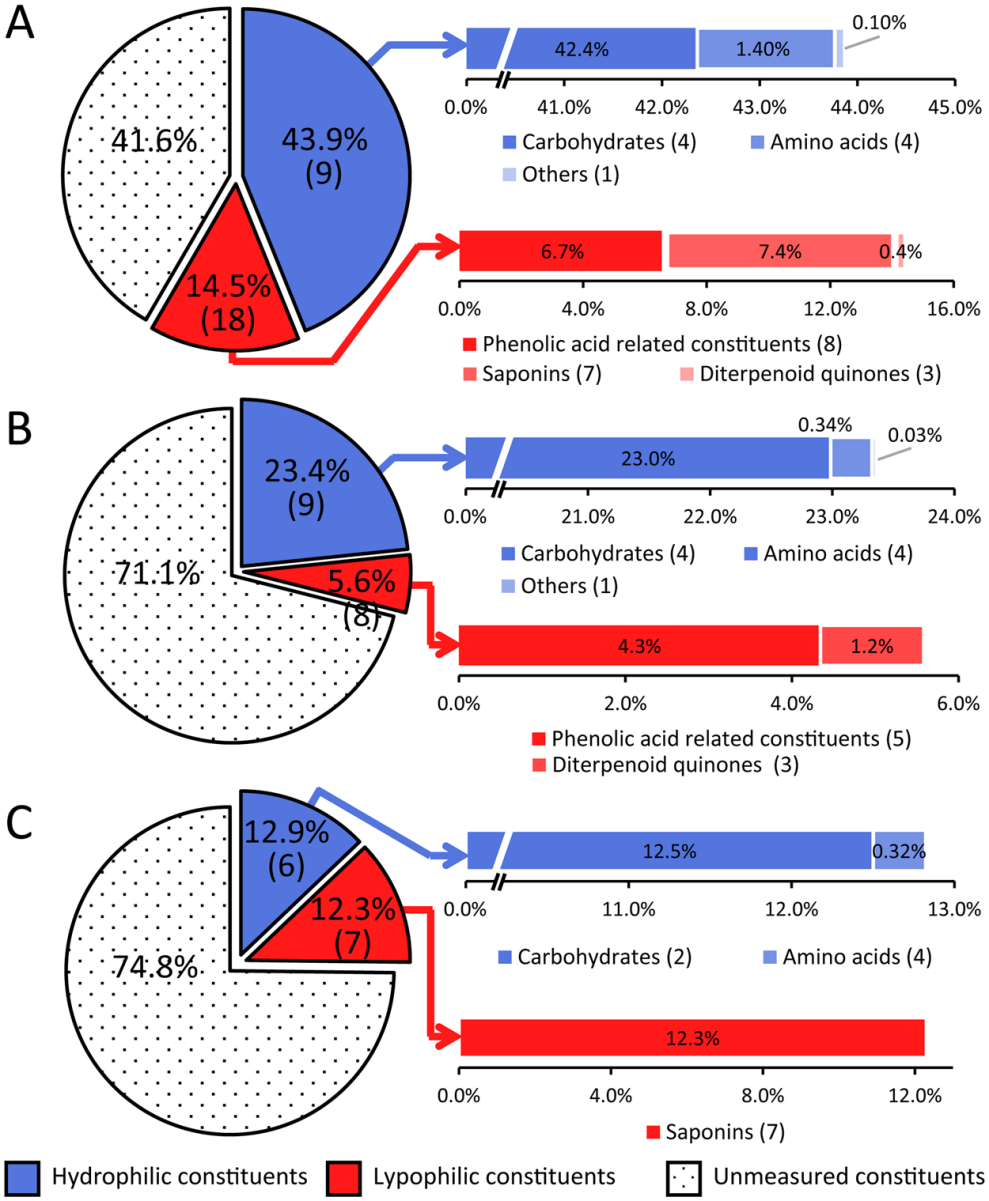
Results of 27 analytes in Dan-Qi Pair extract (**A**), *Radix Salvia miltiorrhiza* (**B**), and *Radix Panax notoginseng* (**C**) using the directly-coupled RP-HILIC-MS/MS system. Average contents of analytes are presented by weight percent. Percentages refer to average contents (by weight percent) in all determined samples. Numbers in the brackets refer to numbers of determined analytes.

The validated method was applied for simultaneous quantitation of 27 analytes in DQP1-DQP30, RSM1-RSM10, and RPN1-RPN10. All results are summarized in Table S4 and Fig. 6. A total of 27, 17, and 13 analytes could be detected in DQP extract, RSM, and RPN, respectively. As expected, no saponin was observed with RSM; accordingly, neither phenolic acid nor diterpenoid quinone was detected in RPN. Overall, the total content from determined analytes in DQP extract, RSM, and RPN were 52.6–69.9%, 21.4–33.9%, and 20.0–30.2%, respectively. Considering that DQP extract also contained about 20% moisture, 6% proteins, 5% tannins, and 5% inorganic salts (data not shown), up to 85–95% constituents in this extract could be revealed. Carbohydrates were the most abundant hydrophilic constituent in all kinds of samples. **C1** and **C2** were determined in all the samples, whereas **C3** and **C4** were only observed in DQP extract (**C3**: 25.21–47.26 mg/g; **C4**: 208.0–341.8 mg/g) and RSM (**C3**: 3.64–14.23 mg/g; **C4**: 52.30–256.8 mg/g). This indicated that **C3** and **C4** originated from RSM. Amino acids were regarded as the second abundant hydrophilic constituents in DQP extract with contents in the range of 0.8–2.1%. Succinic acid accounted for less than 1% of the content in all the samples. Phenolic acids and saponins were two kinds of the main lipophilic constituents that originated from RSM and RPN, respectively. The total contents of determined phenolic acids in DQP extract and RSM were 4.9–8.9% and 1.1–6.4%, respectively; accordingly, those of determined saponins in DQP extract and RPN were 5.4–9.8% and 8.2–15.0%, respectively. It is noteworthy that three phenolic acids, namely **P2**, **P3**, and **P8**, were only detected in DQP extract, agreeing well with the results archived in the literature^43^. These constituents were scarcely present in the raw materials and mainly generated in the process of extraction. Diterpenoid quinones showed lower contents in DQP extract (0.2–0.8%) than RSM (0.7–1.9%). In summary, those diterpenoid quinones, phenolic acids, **O1**, **C3**, and **C4** in DQP extract were derived from RSM, and saponins were originated from RPN, while the other hydrophilic constituents should be supplied by both raw materials.

### Cross-validation of the quantitative results

For the sake of confirming the determined results of the proposed RP-HILIC-MS/MS method, two experiments based conventional quantitative methods were carried out. Based on the separation performances as shown in Fig. 4 and previous studies, RPLC-MS/MS was introduced to confirm the contents of succinic acid, phenolic acids, saponins, and diterpenoid quinones by using the XR-ODS column^34^, whereas HILIC-MS/MS was conducted to assess the contents of amino acids and carbohydrates by employing the amide column^44, 45^. The results obtained by RPLC-MS/MS and HILIC-MS/MS agreed well with the results shown in Table S4 with all the relative errors less than ± 10%. To confirm the necessity of the development of a directly-coupled RP-HILIC-MS/MS system, relative errors between the results supplied by RPLC-MS/MS and HILIC-MS/MS were also calculated. The contents of **A3**, **P1**, **P2**, and all the saponins were in great coincidence between RPLC-MS/MS and HILIC-MS/MS. When utilizing the RPLC-MS/MS system, the determined values of **A4** and all the carbohydrates were significantly increased, whereas those of **A3** obviously decreased. By using the HILIC/MS-MS system, **O1**, **D1**, **D2**, **P7**, and **P8** gave apparently overestimated contents comparing with those obtained from the RPLC-MS/MS system; accordingly, other phenolic acids gave underestimated results. The results showed that conventional quantitative methods could not accurately determine all the analytes in a single run. This was mainly caused by insufficient chromatography separation leaving to serious matrix-induced response enhancement or suppression. Above all, the proposed system is reliable for simultaneously determining the hydrophilic and lipophilic components in DQP extract, RSM and RPN.

It is worth noting that the proposed RP-HILIC-MS/MS system may lose some sensitivities comparing to each single column system. The response ratios of all the analytes in DQP extract using different methods are showed in Fig. S2. RP-MS/MS gave higher responses for **A1**, a few phenolic acids (**P6**–**P7**) and saponins (**S1**, **S4**), and all the internal standards, and HILIC-MS/MS for most carbohydrates (**C1–C3**), saponins (**S1–S5**, **S7**) and internal standards (**IS2**–**IS3**). Among them, the high responses of carbohydrates in RP-MS/MS and internal standards in HILIC-MS/MS could mainly attribute to the matrix enhancement effect induced by the serious coelution at dead time. However, RP-HILIC-MS/MS still processed enough sensitivities to quantitatively analyze all the two medicinal materials and their combination extracts. Further optimization may necessary for samples with trace amounts of analytes (especially saponins).

To date, several methods had been carried on to improve the quality control of DQP related preparations, such as HPLC-DAD-ELSD^35^ and HPLC-TOF-MS^46^. However, these methods are mainly limited to three kinds of constituents (phenolic acids, diterpenoid quinones, and saponins) that could be easily separated in widely used RP-LC. It follows that no more than 16% constituents could be determined in a single run within dozens of minutes. Focusing on these problems, the RP-HILIC-MS/MS approach was proposed. Benefit from the employment of ultra-performance liquid chromatography and tandem mass spectrometry, constituents spanned wide content ranges (>5 orders of magnitude) in DQP extracts could be simultaneously determined in a short time (15 min). Meanwhile, the directly coupling of RP and HILIC significantly expended the polarity ranges (−6.240 ≤ cLogP ≤ 5.471) of the analytes, and conduced to comprehensively understanding the constituents covering most chemical families in DQP extract. Moreover, the new approach served as an in-depth quality evaluation tool of DQP extract by revealing up to 60% content in samples. Collectively, the proposed RP-HILIC-MS/MS is proved to be rapid, comprehensive, and in-depth in evaluating DQP extracts, and was also expected to benefit the controllability of herbal medicines.

## Conclusion

It is important to increase the separation capacity of chromatography for the quantification of multiple hydrophilic and lipophilic constituents in matrices of great complexity. A directly-coupled RP-HILIC-MS/MS system was designed and constructed to solve this issue. In this system, an RP column was serially connected to an HILIC column by using a T-piece to achieve separations based on different mechanisms. A total of 27 analytes with a great polarity span (containing amino acids, carbohydrates, phenolic acids, saponins, diterpenoid quinones, and others) was determined in DQP extract, RSM and RPN. The results expanded our understanding of DQP extract from the aspect of chemical composition as well as mass balance. The present RP-HILIC-MS/MS system is demonstrated to be a reliable tool to benefit the separation and quantification of both hydrophilic and lipophilic constituents in herbal medicines as well as other complicated matrices.

## Material and Methods

### Chemicals, reagents, and materials

MS grade acetonitrile was obtained from Omni Chem (Schaumburg, IL, USA). HPLC grade formic acid (>95% pure) was from Sigma (Saint Louis, MO, USA). High purity deionized water was purified by a Milli-Q system (Bedford, MA, USA). Reference standards of salvianolic acid D (**P3**), lithospermic acid (**P5**), salvianolic acid A (**P6**), salvianolic acid C (**P8**), ginsenoside Rg_2_ (**S5**), and ginsenoside Rh_1_ (**S6**) were purchased from Yifang Technology Co., Ltd., (Tianjin, China). *α*-D-glucopyranosyl-(1→2)-*β*-D-fructofuranosyl-(1→1)-*α*-D-galactopyranose (**C3**) and D-stachyose (**C4**) were supplied by Tianjin Tasly Modern TCM Resources Co. Ltd (Tianjin, China). L-lysine-4,4,5,5-*d*_4_ (**IS1**) was purchased from Sigma. Other standards were purchased from the National Institute for Control of Biological and Pharmaceutical (Beijing, China).

DQP extracts (DQP1-DQP30) that derived from RSM and RPN by using water extraction and alcohol precipitation were provided by Tianjin Tasly Pharmaceutical Co. Ltd (Tianjin, China). Dried medicinal materials, including RSM (RSM1-RSM10) and RPN (RPN1-RPN10) samples, were collected from Shaanxi and Yunnan provinces in China, respectively. Only dry treatment was preformed to remove the moisture in raw materials after harvest. Voucher specimens were deposited at Tasly Academy.

### Preparation of standard solutions

The standard solutions were prepared by dissolving the appropriate amount of each reference standard with 70% methanol in a 10 mL volumetric flask. Subsequently, the mixed stock solution was prepared by mixing all the standard solutions and diluting with 70% methanol in a 10 mL volumetric flask. Finally, a series of solutions was consecutively diluted with the stock solution using 20% aqueous methanol fortified with **IS1**, **IS2**, and **IS3** (50 ng/mL for each) to prepare the standard solutions for calibration (dilution factor = 2, 5, 10, 20, 50, 100, 200, 500, 1000). All the solutions were stored in a refrigerator at 4 °C prior to analysis.

### Preparation of sample solutions

Extract of DQP, powdered RSM, and powdered RPN samples of 0.10 g were accurately weighted. All were sonicated in 100 mL of 70% methanol for 30 min using a KQ-500DE ultrasonic bath (120 W, 40 kHz) (Shanghai, China). After cooling to room temperature, the supernatant was collected and filtered through a membrane filter (0.22 μm). For quantitative analysis, an aliquot (50 μL) of each filtrate was 40-fold diluted with 70% aqueous methanol containing **IS1**, **IS2**, and **IS3** (50 ng/mL for either). All the solutions were stored in refrigerator at 4 °C prior to analysis.

### RP-HILIC-MS/MS analysis

Sample separations were performed on a Shimadzu LC-30 system, comprised four solvent delivery pumps, an on-line degasser, an auto-sampler, and a column temperature controller (Shimadzu, Tokyo, JP). RPLC separation was performed on two solvent delivery pumps (pump A_1_ and B_1_) equipped with a Shim-Pack XR-ODS III column (1.6 μm particles, 2.1 mm i.d. × 50 mm) and an on-line filter. HILIC separation was carried out utilizing two solvent delivery pumps (pump A_2_ and B_2_) equipped with a Waters Acquity UPLC® BEH Amide column (1.7 μm particles, 2.1 mm i.d. × 100 mm, Waters, MA, USA). The XR-ODS and BEH Amide column were directly-coupled in series using a zero-volume T-piece connector. The inlet of the RP column was connected to the autosampler and the third port of the T-piece was connected to pump A_2_ and B_2_. The injection volume was 2 μL and the column oven was set at 30 °C. The mobile phase was composed of 0.1% aqueous formic acid (phase A) and acetonitrile (phase B). The flow rates of RPLC and HILIC were set at 0.12 mL/min and 0.40 mL/min, respectively. The RPLC mobile phase was composed of A_1_ and B_1_ using a gradient elution of 25% B at 0 min, 25% B at 0.5 min, 35% B at 1.0 min, 60% B at 5.0 min, 80% B at 10.0 min, and 80% at 15.0 min, while that of HILIC was composed of A_2_ and B_2_ using a gradient elution of 97% B at 0 min, 50% B at 10.0 min, 50% B at 10.5 min, 97% B at 11.0 min, and 97% B at 15.0 min. The eluent of the RPLC-HILIC was diverted to waste during the first 1.5 min to prevent contamination of the MS system.

An Applied Biosystems Sciex Qtrap® 5500 MS/MS system (Foster, CA, USA) equipped with an electrospray ionization source was employed as the detector. MS conditions were set as follows: positive ion-spray voltage, 5500 V; negative ion-spray voltage, −4500 V; ion source temperature, 550 °C; nebulizer gas (gas 1, nitrogen) and heater gas (gas 2, nitrogen), 55 psi; curtain gas, 25 psi; interface heater, on. The quantitative analysis of all analytes was performed using the schedule multiple reaction monitoring (MRM) method as shown in Table 1. The detection time window for each ion transition was set as 60 s (retention time ± 30 s). Data acquisition and processing were carried out using Analyst® software (Version 1.6.2). The smoothing factor was set as 2 and the bunching factor as 1 for all monitored peaks.

### Validation and evaluation of PR-HILIC-MS/MS

The method was validated based on USP 36 〈1225 VALIDATION OF COMPENDIAL PROCEDURES〉, including selectivity, linearity, accuracy, precision, limit of detection, lower limit of quantification, and stability. After validation, the method was applied to simultaneously determine 9 hydrophilic and 18 lipophilic components in the extracts of DQP (DQP1-DQP30), RSM (RSM1-RSM10), and RPN (RPN1-RPN10). The results obtained from RP-HILIC-MS/MS were compared with those from conventional method, namely RPLC-MS/MS and HILIC-MS/MS.

## Electronic supplementary material

Supplementary information
