# Supplementary material for: Simultaneous determination of hydrophilic and lipophilic constituents in herbal medicines using directly-coupled reversed-phase and hydrophilic interaction liquid chromatography-tandem mass spectrometry
Source: Sci Rep. 2017 Aug 1;7:7061. doi: 10.1038/s41598-017-07087-x (PMC5539142; doi:10.1038/s41598-017-07087-x)
Supplement: Supplementary file 1 — Supplementary information [file 41598_2017_7087_MOESM1_ESM.pdf]

Supplemental Information

Simultaneous determination of hydrophilic and lipophilic constituents in herbal medicines using directly-coupled reversed-phase and hydrophilic interaction liquid chromatography-tandem mass spectrometry

Wan-Yang Sun[a,b,1], Qin-Wei Lu[c,1], Hao Gao[a,*], Ling Tong[d], Dong-Xiang Li[d], Zheng-Qun Zhou[a], Zheng-Jin Jiang[e], Henry Sun[d], Kai-Shun Bi[b,*]

[a] *Institute of Traditional Chinese Medicine &Natural Products, College of Pharmacy, Jinan University, Guangzhou, Guangdong 510632, China*
[b] *National and Local Joint Engineering Laboratory for Key Technology of Chinese Material Medica Quality Control, School of Pharmacy, Shenyang Pharmaceutical University, Shenyang 110016, China*
[c] *School of Pharmacy, China Pharmaceutical University, Nanjing 210009, China*
[d] *State Key Laboratory of Core Technology in Innovative Chinese Medicine, Pharmaceutical Analysis Institute, Tasly Academy, Tianjin 300402, China*
[e] *Department of Pharmacy and Guangdong Province Key Laboratory of Pharmacodynamic Constituents of Traditional Chinese Medicine & New Drug Research, Jinan University, Guangzhou 510632, China*

* Corresponding author at: School of Pharmacy, Jinan University, Guangzhou 510632, China
Tel.: +86 20 85228369; fax: +86 20 85228369.
E-mail address: tghao@jnu.edu.cn (H. Gao).

* Corresponding author at: School of Pharmacy, Shenyang Pharmaceutical University, Shenyang 110016, China
Tel.: +86 24 23986012; fax: +86 24 23986012.
E-mail address: kaishunbi.syphu@gmail.com (K.S. Bi).

[1]These authors contributed equally to this work.

Content

Figure S1 Extraction efficiency of 27 analytes. Heat map plot shows the normalized extract efficiency of each analytes. The analyte abbreviations are given in Table 1.

Figure S2 The response ratios of 27 analytes in Dan-Qi pair extract among RP-HILIC-MS/MS, RP-MS/MS, and HILIC-MS/MS.

Table S1 Internal standards, linear regression data, limits of quantification (LODs), and limits of detection (LOQs) for 27 analytes.

Table S2 Precision, repeatability and stability for 27 analytes in Dan-Qi pair extract, *Raxid Salvia miltiorrhiza* and *Raxid Panax notoginseng*.

Table S3 Recovery for 27 analytes in Dan-Qi pair extract, *Raxid Salvia miltiorrhiza* and *Raxid Panax notoginseng* (RSD, %, *n*=3).

Table S4 Results for Dan-Qi pair extract (DQP1-DQP30), *Raxid Salvia miltiorrhiza* (RSM1-RSM10) and *Raxid Panax notoginseng* (RPN1-RPN10) (mg/g).

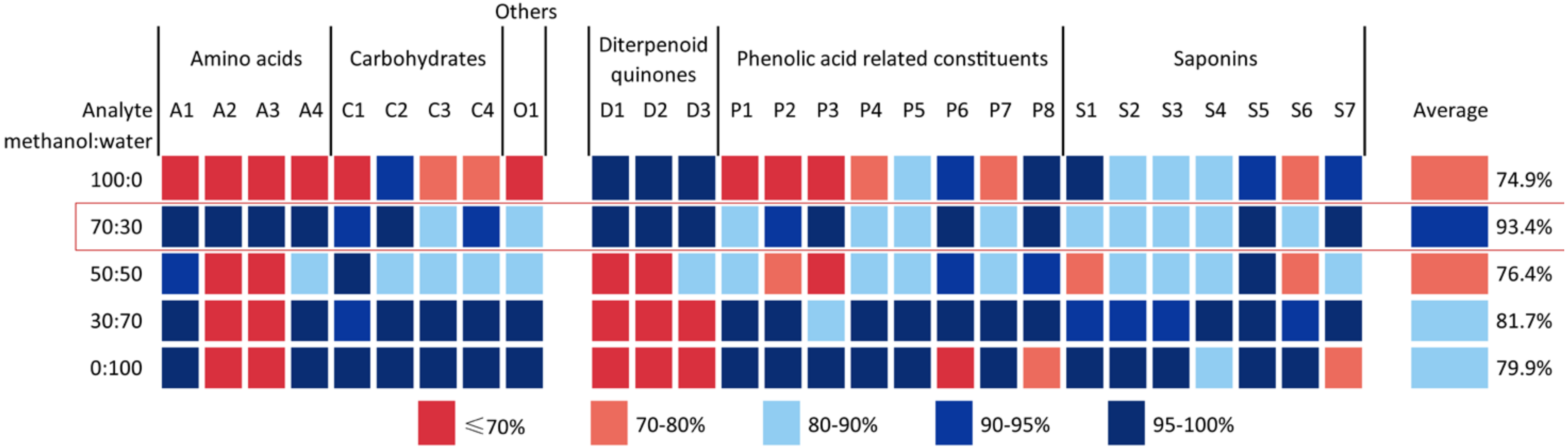

Figure S1 Extraction efficiency of 27 analytes. Heat map plot shows the normalized extract efficiency of each analytes. The analyte abbreviations are given in Table 1.

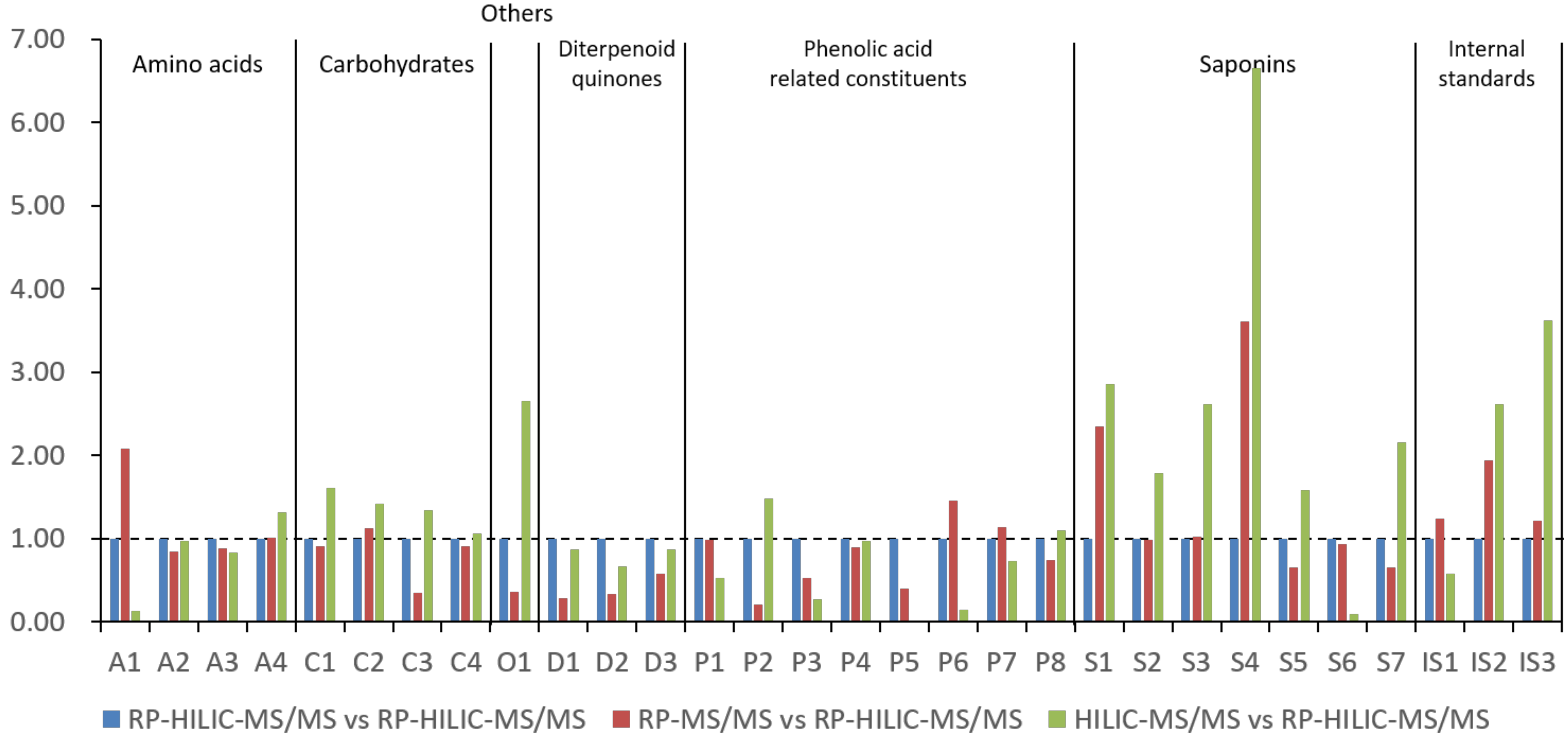

Figure S2 The response ratios of 27 analytes in Dan-Qi pair extract among RP-HILIC-MS/MS, RP-MS/MS, and HILIC-MS/MS.

Table S1 Internal standards, linear regression data, limits of quantification (LODs), and limits of detection (LOQs) for 27 analytes.

NO.	Internal standard	Regression equation	r^2	Range (ng/mL)	LOQ (ng/mL)	LOD (ng/mL)
A1	Chloramphenicol	$y = 9.9 \times 10^{-4}\ x + 4.33 \times 10^{-2}$	0.9975	2.463 - 985.0	1.072	0.482
A2	L-lysine-4,4,5,5-d_4	$y = 1.56 \times 10^{-1}\ x + 5.47 \times 10^{-1}$	0.9952	0.254 - 254.0	0.128	0.063
A3	L-lysine-4,4,5,5-d_4	$y = 1.97 \times 10^{-2}\ x + 2.36 \times 10^{-1}$	0.9991	0.506 - 506.0	0.253	0.122
A4	L-lysine-4,4,5,5-d_4	$y = 4.45 \times 10^{-2}\ x + 1.73 \times 10^{-1}$	0.9991	0.254 - 254.0	0.204	0.088
C1	Chloramphenicol	$y = 7.66 \times 10^{-3}\ x + 1.44 \times 10^{-1}$	0.9979	2.990 - 2.990 × 10^3	1.935	0.416
C2	Chloramphenicol	$y = 3.13 \times 10^{-2}\ x + 1.31$	0.9936	25.12 - 2.512 × 10^4	3.313	1.047
C3	Chloramphenicol	$y = 9.0 \times 10^{-3}\ x + 2.29 \times 10^{-2}$	0.9987	1.008 - 1.008 × 10^3	1.008	0.319
C4	Chloramphenicol	$y = 4.51 \times 10^{-3}\ x + 9.8 \times 10^{-2}$	0.9993	34.75 - 3.475 × 10^4	2.748	0.851
D1	Diazepam	$y = 7.17 \times 10^{-1}\ x - 6.59 \times 10^{-2}$	0.9975	0.489 - 244.5	0.298	0.109
D2	Diazepam	$y = 2.20 \times 10^{-1}\ x - 3.37 \times 10^{-2}$	0.9960	0.520 - 260.0	0.231	0.117
D3	Diazepam	$y = 1.67 \times 10^{-3}\ x - 2.37 \times 10^{-1}$	0.9993	1.765 - 882.5	1.268	0.399
O1	Chloramphenicol	$y = 1.65 \times 10^{-1}\ x + 6.96 \times 10^{-1}$	0.9993	0.257 - 128.5	0.152	0.731
P1	Chloramphenicol	$y = 3.11 \times 10^{-2}\ x + 4.56 \times 10^{-2}$	0.9991	2.475 - 495.0	1.560	0.302
P2	Chloramphenicol	$y = 9.7 \times 10^{-1}\ x + 2.10 \times 10^{-1}$	0.9983	0.249 - 248.5	0.195	0.083
P3	Chloramphenicol	$y = 6.16 \times 10^{-2}\ x - 3.02 \times 10^{-2}$	0.9991	0.492 - 246.0	0.289	0.083
P4	Chloramphenicol	$y = 1.41 \times 10^{-1}\ x + 3.78 \times 10^{-2}$	0.9990	0.975 - 487.5	0.781	0.216
P5	Chloramphenicol	$y = 1.41 \times 10^{-1}\ x + 8.1 \times 10^{-3}$	0.9988	2.525 - 505.0	1.250	0.521
P6	Chloramphenicol	$y = 1.46 \times 10^{-1}\ x - 1.06 \times 10^{-1}$	0.9996	2.605 - 521.0	1.173	0.518
P7	Chloramphenicol	$y = 2.62 \times 10^{-2}\ x + 2.80 \times 10^{-2}$	0.9993	5.940 - 5.940 × 10^3	1.176	0.217
P8	Chloramphenicol	$y = 9.2 \times 10^{-2}\ x + 6.54 \times 10^{-2}$	0.9988	0.488 - 244.0	0.350	0.130
S1	Chloramphenicol	$y = 2.28 \times 10^{-3}\ x + 1.85 \times 10^{-2}$	0.9988	2.475 - 990.0	1.859	0.840
S2	Chloramphenicol	$y = 1.01 \times 10^{-3}\ x + 4.44 \times 10^{-3}$	0.9993	4.040 - 4.040 × 10^3	2.977	0.891
S3	Chloramphenicol	$y = 6.22 \times 10^{-4}\ x + 7.76 \times 10^{-3}$	0.9995	2.445 - 489.0	1.025	0.333
S4	Chloramphenicol	$y = 2.21 \times 10^{-4}\ x - 2.14 \times 10^{-3}$	0.9988	20.150 - 2.015 × 10^3	3.382	1.017
S5	Chloramphenicol	$y = 1.15 \times 10^{-2}\ x + 1.29 \times 10^{-4}$	0.9995	1.288 - 515.0	0.715	0.226
S6	Chloramphenicol	$y = 6.61 \times 10^{-4}\ x + 1.85 \times 10^{-3}$	0.9976	1.255 - 502.0	0.626	0.219
S7	Chloramphenicol	$y = 4.34 \times 10^{-4}\ x - 1.35 \times 10^{-3}$	0.9984	2.021 - 2.021 × 10^3	0.529	0.103

Table S2 Recovery for 27 analytes in Dan-Qi pair, *Raxid Salvia miltiorrhiza* and *Raxid Panax notoginseng* (RSD, %, *n*=3).

NO.	Extract of Dan-Qi pair			*Radix Salvia miltiorrhiza*			*Radix Panax notoginseng*		
	Low	Medium	High	Low	Medium	High	Low	Medium	High
A1	101.8 (1.2)	98.6 (2.8)	100.5 (0.9)	98.9 (2.0)	103.7 (2.4)	98.9 (1.9)	101.1 (1.2)	97.6 (2.7)	102.4 (1.4)
A2	98.6 (3.0)	103.4 (2.3)	99.4 (1.4)	101.8 (1.9)	98.6 (2.0)	99.7 (2.4)	96.6 (2.1)	101.1 (1.9)	97.9 (2.8)
A3	98.4 (0.9)	98.1 (1.4)	100.8 (2.2)	102.3 (2.0)	101.7 (1.9)	97.7 (2.1)	103.1 (2.0)	102.8 (2.0)	100.8 (1.4)
A4	103.5 (2.4)	98.8 (2.4)	103.8 (2.5)	97.9 (2.7)	104.0 (2.6)	102.7 (2.5)	97.9 (2.6)	101.4 (2.1)	103.0 (2.4)
C1	102.0 (4.3)	101.7 (2.6)	102.0 (3.0)	106.7 (2.9)	100.8 (4.0)	97.3 (2.8)	104.5 (1.8)	96.0 (2.5)	100.8 (2.0)
C2	97.4 (2.4)	101.8 (1.8)	102.8 (3.1)	100.0 (2.7)	97.5 (2.1)	96.1 (2.8)	96.6 (1.5)	97.0 (2.2)	101.3 (2.9)
C3	101.8 (1.9)	98.9 (1.7)	96.6 (2.1)	101.0 (2.2)	97.7 (1.7)	96.8 (1.9)	-	-	-
C4	97.8 (1.3)	98.3 (1.8)	100.8 (2.2)	100.8 (2.3)	96.1 (2.2)	95.6 (1.4)	-	-	-
D1	96.0 (4.9)	100.2 (2.8)	102.9 (1.9)	101.8 (4.6)	101.8 (3.8)	106.4 (3.4)	-	-	-
D2	98.6 (2.2)	100.5 (2.2)	103.8 (1.0)	100.5 (2.6)	99.8 (1.8)	101.3 (2.2)	-	-	-
D3	99.7 (4.1)	95.1 (4.1)	96.3 (2.6)	103.3 (5.3)	101.9 (4.3)	96.2 (4.0)	-	-	-
O1	97.8 (4.1)	102.3 (3.0)	101.7 (2.1)	97.3 (3.8)	99.8 (2.7)	104.0 (3.2)	-	-	-
P1	97.9 (0.9)	105.0 (1.8)	102.2 (1.0)	100.1 (2.4)	102.2 (0.9)	100.5 (2.3)	-	-	-
P2	97.9 (3.9)	98.5 (4.7)	100.8 (4.3)	-	-	-	-	-	-
P3	100.5 (1.9)	103.4 (0.9)	100.6 (1.6)	-	-	-	-	-	-
P4	99.8 (2.6)	100.6 (2.5)	104.1 (1.2)	97.9 (2.2)	98.6 (1.5)	101.2 (1.8)	-	-	-
P5	99.8 (3.5)	102.1 (2.4)	100.8 (1.0)	100.5 (1.2)	100.5 (1.0)	103.0 (1.8)	-	-	-
P6	99.2 (3.3)	103.4 (2.5)	103.7 (1.6)	96.0 (2.9)	99.2 (2.0)	101.1 (1.9)	-	-	-
P7	101.1 (4.6)	101.8 (3.2)	100.8 (3.9)	100.3 (4.6)	101.6 (3.6)	103.5 (2.7)	-	-	-
P8	97.9 (2.3)	100.8 (1.3)	99.8 (1.5)	-	-	-	-	-	-
S1	101.8 (2.7)	100.8 (1.3)	98.4 (0.9)	-	-	-	100.5 (3.0)	100.2 (1.3)	98.7 (1.3)
S2	101.1 (3.3)	98.2 (3.1)	102.4 (1.7)	-	-	-	101.1 (4.1)	97.0 (4.2)	98.4 (2.7)
S3	100.5 (2.9)	102.7 (2.7)	103.7 (1.0)	-	-	-	101.1 (2.0)	100.5 (2.3)	101.2 (1.7)
S4	97.6 (3.0)	96.0 (3.0)	106.8 (4.4)	-	-	-	96.0 (2.8)	99.2 (3.3)	97.6 (3.8)
S5	99.7 (5.0)	99.5 (1.2)	94.2 (1.9)	-	-	-	99.2 (2.8)	103.2 (4.4)	102.0 (2.1)
S6	98.7 (3.3)	100.1 (1.6)	97.4 (1.9)	-	-	-	99.2 (2.8)	100.8 (4.6)	99.2 (2.7)
S7	96.6 (3.5)	101.4 (3.0)	97.0 (2.3)	-	-	-	100.9 (1.8)	97.4 (1.7)	98.7 (2.0)

*Dan-Qi pair (DQP01); *Raxid Salvia miltiorrhiza* (RSM07); *Raxid Panax notoginseng* (RPN06).

Table S3 Precision, repeatability and stability for 27 analytes in Dan-Qi pair, *Raxid Salvia miltiorrhiza* and *Raxid Panax notoginseng*.*

NO.	Dan-Qi pair				*Radix Salvia miltiorrhiza*				*Radix Panax notoginseng*			
	Precision		Repeatability RSD (%) (*n*=6)	Stability RSD (%)	Precision		Repeatability RSD (%) (*n*=6)	Stability RSD (%)	Precision		Repeatability RSD (%) (*n*=6)	Stability RSD (%)
	Intra-day (*n*=6)	Inter-day (*n*=3)			Intra-day (*n*=6)	Inter-day (*n*=3)			Intra-day (*n*=6)	Inter-day (*n*=3)		
A1	1.7	3.5	2.9	2.5	2.6	2.3	3.4	3.4	2.4	3.1	1.1	4.3
A2	0.6	2.2	3.6	3.5	2.3	3.2	3.3	4.2	1.3	2.7	3.5	4.0
A3	1.3	3.6	4.4	2.4	2.6	4.4	3.9	2.6	2.2	4.8	4.5	2.1
A4	1.5	3.5	4.6	2.0	3.1	2.5	1.9	3.7	3.2	4.0	2.0	3.6
C1	1.4	4.4	2.6	4.8	2.3	4.7	3.0	4.4	1.8	2.3	4.8	2.6
C2	0.7	1.3	2.1	2.3	1.3	1.3	2.9	2.5	2.3	4.9	3.9	3.1
C3	2.3	3.6	3.9	3.2	1.4	1.8	1.9	2.0	-	-	-	-
C4	1.9	2.8	4.5	4.7	0.7	1.7	1.7	3.7	-	-	-	-
D1	2.8	3.3	4.2	4.7	3.0	4.6	2.7	3.5	-	-	-	-
D2	3.1	2.5	3.2	3.7	2.0	4.9	3.2	2.6	-	-	-	-
D3	1.8	4.3	3.4	3.6	3.3	4.5	4.6	3.0	-	-	-	-
O1	1.9	2.3	1.9	4.6	2.5	1.7	2.1	3.4	-	-	-	-
P1	2.7	3.1	3.5	3.3	2.9	4.7	2.9	3.3	-	-	-	-
P2	2.8	3.4	2.2	4.0	-	-	-	-	-	-	-	-
P3	2.0	4.9	3.8	4.2	-	-	-	-	-	-	-	-
P4	2.2	3.6	4.8	2.7	1.5	2.0	2.9	3.4	-	-	-	-
P5	2.4	3.6	3.8	3.0	1.9	4.9	5.0	3.5	-	-	-	-
P6	2.7	3.7	3.9	1.6	2.0	5.4	2.3	4.0	-	-	-	-
P7	1.9	2.5	1.6	4.1	2.3	4.8	2.1	4.4	-	-	-	-
P8	2.0	4.2	3.4	4.6	-	-	-	-	-	-	-	-
S1	3.3	4.7	4.8	3.4	-	-	-	-	2.1	4.7	3.4	4.3
S2	4.9	4.4	4.4	4.4	-	-	-	-	2.5	3.1	3.2	3.0
S3	1.3	3.6	4.4	4.9	-	-	-	-	2.2	3.2	3.6	3.6
S4	2.5	4.1	4.3	3.9	-	-	-	-	1.7	3.2	5.6	3.3
S5	2.4	3.7	4.6	2.7	-	-	-	-	1.2	2.9	4.8	2.3
S6	3.5	4.8	2.0	2.3	-	-	-	-	1.5	2.2	3.2	3.0
S7	2.5	2.6	4.9	4.2	-	-	-	-	1.4	2.5	2.3	3.9

*Dan-Qi pair (DQP01); *Raxid Salvia miltiorrhiza* (RSM07); *Raxid Panax notoginseng* (RPN06).

Table S4 Results for Dan-Qi pair (DQP1-DQP30), *Raxid Salvia miltiorrhiza* (RSM1-RSM10) and *Raxid Panax notoginseng* (RPN1-RPN10) (mg/g).

Sample	A1[a]	A2	A3	A4	C1	C2	C3	C4	D1	D2
DQP1	9.90 ± 2.10	0.224 ± 0.012	2.285 ± 0.185	1.885 ± 0.181	23.87 ± 0.66	67.94 ± 0.31	34.68 ± 1.87	255.5 ± 11.5	0.605 ± 0.011	0.514 ± 0.002
DQP2	7.607 ± 0.532	0.441 ± 0.104	6.922 ± 2.263	2.918 ± 0.013	24.57 ± 1.70	77.37 ± 2.44	37.80 ± 4.21	253.3 ± 18.9	0.492 ± 0.023	1.274 ± 0.015
DQP3	11.27 ± 0.59	0.220 ± 0.005	2.488 ± 0.417	3.397 ± 0.176	21.43 ± 2.51	85.8 ± 7.9	25.21 ± 0.94	240.5 ± 28.9	1.832 ± 0.212	1.826 ± 0.393
DQP4	11.67 ± 1.38	0.174 ± 0.008	1.832 ± 0.106	3.074 ± 0.221	32.39 ± 1.77	106.9 ± 0.2	47.06 ± 0.68	331.9 ± 25.7	2.265 ± 0.148	2.170 ± 0.165
DQP5	6.332 ± 0.473	0.029 ± 0.001	0.272 ± 0.002	1.234 ± 0.050	26.86 ± 1.94	70.65 ± 8.06	33.38 ± 3.53	298.9 ± 17.5	1.157 ± 0.043	0.836 ± 0.016
DQP6	8.62 ± 0.89	0.143 ± 0.009	1.805 ± 0.274	2.321 ± 0.038	29.77 ± 2.89	75.92 ± 3.94	39.03 ± 0.30	331.6 ± 39.2	1.275 ± 0.107	1.559 ± 0.069
DQP7	9.31 ± 0.42	0.317 ± 0.087	3.729 ± 0.168	1.050 ± 0.233	24.18 ± 0.06	52.79 ± 4.39	40.81 ± 4.24	269.1 ± 10.7	0.477 ± 0.004	1.407 ± 0.128
DQP8	10.85 ± 0.74	0.062 ± 0.002	1.011 ± 0.312	3.334 ± 0.116	23.37 ± 3.53	80.9 ± 2.2	29.40 ± 0.43	240.2 ± 50.4	0.575 ± 0.006	1.079 ± 0.100
DQP9	8.29 ± 1.04	0.307 ± 0.089	1.882 ± 0.449	1.869 ± 0.260	28.28 ± 1.15	91.4 ± 3.7	44.57 ± 1.44	288.2 ± 12.2	0.611 ± 0.001	0.941 ± 0.125
DQP10	10.58 ± 0.57	0.199 ± 0.010	2.677 ± 0.037	3.092 ± 0.036	22.46 ± 3.07	84.5 ± 2.5	31.53 ± 0.01	250.8 ± 48.5	0.836 ± 0.106	1.488 ± 0.136
DQP11	6.568 ± 0.269	0.368 ± 0.004	2.272 ± 0.035	3.984 ± 0.167	26.72 ± 1.36	88.4 ± 0.4	34.00 ± 0.53	278.4 ± 21.4	0.592 ± 0.002	1.238 ± 0.031
DQP12	6.494 ± 0.020	0.149 ± 0.053	1.882 ± 0.248	2.551 ± 0.466	29.97 ± 0.26	77.47 ± 1.15	36.86 ± 1.50	335.2 ± 6.5	1.275 ± 0.023	1.786 ± 0.139
DQP13	8.62 ± 0.72	0.359 ± 0.007	1.577 ± 0.114	2.128 ± 0.041	25.64 ± 1.17	79.23 ± 12.02	35.82 ± 1.42	269.5 ± 4.0	0.795 ± 0.228	1.589 ± 0.058
DQP14	5.620 ± 1.108	0.239 ± 0.005	1.644 ± 0.309	2.003 ± 0.173	19.68 ± 3.25	98.1 ± 4.0	28.43 ± 1.74	231.0 ± 34.0	1.117 ± 0.125	1.248 ± 0.016
DQP15	13.44 ± 0.01	0.216 ± 0.009	2.869 ± 0.175	4.053 ± 0.216	26.42 ± 3.76	79.72 ± 3.78	36.53 ± 1.35	280.0 ± 54.0	1.429 ± 0.062	1.505 ± 0.177
DQP16	7.402 ± 0.331	0.256 ± 0.050	2.663 ± 0.111	2.994 ± 0.010	29.68 ± 1.22	89.4 ± 0.8	36.99 ± 3.69	318.9 ± 15.5	0.601 ± 0.006	1.390 ± 0.003
DQP17	9.76 ± 1.83	0.122 ± 0.018	0.922 ± 0.088	3.937 ± 0.268	27.49 ± 1.67	101.8 ± 0.7	39.84 ± 2.20	270.8 ± 23.5	0.612 ± 0.029	0.977 ± 0.122
DQP18	5.584 ± 0.583	0.088 ± 0.001	1.474 ± 0.108	1.818 ± 0.015	21.45 ± 1.09	87.9 ± 6.6	29.72 ± 4.65	293.0 ± 24.1	1.204 ± 0.061	0.911 ± 0.087
DQP19	11.18 ± 2.19	0.184 ± 0.013	0.927 ± 0.177	1.843 ± 0.107	33.24 ± 1.37	110.1 ± 8.4	46.76 ± 0.72	341.8 ± 12.9	0.471 ± 0.027	1.443 ± 0.071
DQP20	9.41 ± 0.30	0.079 ± 0.004	1.149 ± 0.102	2.811 ± 0.565	29.73 ± 1.24	141.5 ± 12.6	33.29 ± 0.68	271.1 ± 30.6	0.706 ± 0.010	0.682 ± 0.063
DQP21	6.834 ± 0.167	0.352 ± 0.090	1.659 ± 0.731	1.828 ± 0.152	20.16 ± 5.79	100.4 ± 1.9	44.68 ± 8.62	208.0 ± 28.0	0.747 ± 0.065	0.695 ± 0.077
DQP22	6.550 ± 0.680	0.262 ± 0.010	1.328 ± 0.075	3.053 ± 0.126	27.52 ± 7.44	92.1 ± 7.6	36.22 ± 6.69	284.5 ± 97.3	0.563 ± 0.003	1.447 ± 0.008
DQP23	9.71 ± 0.85	0.295 ± 0.034	1.477 ± 0.324	1.945 ± 0.069	24.80 ± 1.04	81.6 ± 4.5	39.19 ± 1.09	251.1 ± 19.1	1.946 ± 0.101	2.186 ± 0.100
DQP24	7.885 ± 0.914	0.240 ± 0.003	1.558 ± 0.070	1.549 ± 0.072	23.25 ± 2.54	60.07 ± 1.22	34.02 ± 1.54	254.7 ± 38.4	1.044 ± 0.081	2.264 ± 0.078
DQP25	10.70 ± 1.31	0.231 ± 0.015	1.442 ± 0.071	2.470 ± 0.086	29.34 ± 0.91	68.40 ± 1.52	47.26 ± 5.31	324.5 ± 20.5	0.644 ± 0.084	1.306 ± 0.101
DQP26	14.01 ± 0.16	0.289 ± 0.020	3.128 ± 0.058	3.344 ± 0.232	25.10 ± 0.88	111.4 ± 14.9	29.93 ± 3.13	235.1 ± 4.8	1.679 ± 0.075	1.045 ± 0.092
DQP27	6.292 ± 1.084	0.090 ± 0.011	0.639 ± 0.102	1.166 ± 0.067	24.19 ± 0.86	60.58 ± 0.56	33.82 ± 2.35	276.2 ± 12.8	1.663 ± 0.031	1.039 ± 0.139
DQP28	11.06 ± 0.70	0.228 ± 0.001	1.884 ± 0.299	3.113 ± 0.344	22.95 ± 3.23	65.75 ± 1.52	36.81 ± 3.21	241.6 ± 9.7	0.877 ± 0.042	1.248 ± 0.210
DQP29	12.90 ± 1.49	0.313 ± 0.011	3.190 ± 0.014	3.278 ± 0.049	25.65 ± 1.30	111.8 ± 9.7	29.83 ± 3.44	243.1 ± 51.3	0.545 ± 0.033	0.441 ± 0.060
DQP30	8.42 ± 0.74	0.431 ± 0.010	4.409 ± 0.094	4.632 ± 0.072	27.28 ± 3.87	60.85 ± 3.84	34.51 ± 4.78	313.8 ± 49.5	1.148 ± 0.120	1.222 ± 0.099
RSM1	0.794 ± 0.123	0.066 ± 0.004	0.673 ± 0.081	0.448 ± 0.063	3.749 ± 0.430	132.7 ± 20.6	3.644 ± 0.579	52.30 ± 7.86	1.396 ± 0.220	2.443 ± 0.275
RSM2	tr.[b]	0.070 ± 0.002	0.475 ± 0.074	1.928 ± 0.202	1.887 ± 0.157	29.54 ± 3.95	12.45 ± 0.25	219.4 ± 21.4	1.494 ± 0.178	2.497 ± 0.206
RSM3	tr.	0.126 ± 0.007	0.554 ± 0.015	1.884 ± 0.149	tr.	23.40 ± 1.33	9.33 ± 0.37	154.5 ± 19.5	3.697 ± 0.566	3.877 ± 0.142
RSM4	tr.	0.035 ± 0.000	0.377 ± 0.002	3.108 ± 0.002	2.050 ± 0.134	25.78 ± 3.72	14.23 ± 1.31	256.8 ± 17.0	1.145 ± 0.099	1.470 ± 0.039
RSM5	tr.	0.065 ± 0.007	0.614 ± 0.094	1.432 ± 0.001	0.593 ± 0.039	18.49 ± 0.93	13.20 ± 1.51	208.4 ± 24.7	3.489 ± 0.467	3.677 ± 0.199
RSM6	1.659 ± 0.243	0.240 ± 0.038	1.200 ± 0.036	0.640 ± 0.016	0.903 ± 0.043	36.91 ± 1.57	12.13 ± 1.57	206.4 ± 16.7	1.925 ± 0.360	2.517 ± 0.149
RSM7	0.496 ± 0.012	0.249 ± 0.008	1.279 ± 0.097	2.128 ± 0.025	1.461 ± 0.062	29.15 ± 2.99	11.18 ± 0.93	210.8 ± 33.0	1.290 ± 0.176	1.748 ± 0.096
RSM8	0.730 ± 0.063	0.153 ± 0.024	0.705 ± 0.018	2.344 ± 0.086	2.300 ± 0.277	25.42 ± 4.07	9.25 ± 0.53	140.8 ± 17.4	2.681 ± 0.521	2.728 ± 0.098
RSM9	tr.	nd.	tr.	1.800 ± 0.337	0.613 ± 0.110	25.98 ± 1.79	8.89 ± 1.43	169.5 ± 23.1	4.273 ± 0.675	3.265 ± 0.023
RSM10	0.318 ± 0.018	0.458 ± 0.021	2.578 ± 0.382	4.280 ± 0.304	3.980 ± 0.414	44.35 ± 6.58	12.29 ± 1.74	164.0 ± 15.5	2.321 ± 0.078	2.932 ± 0.219
RPN1	2.372 ± 0.071	0.200 ± 0.003	0.519 ± 0.013	3.560 ± 0.207	12.97 ± 0.85	133.5 ± 14.0	nd.	nd.	nd.	nd.
RPN2	tr.	0.032 ± 0.007	0.262 ± 0.026	2.304 ± 0.316	19.62 ± 0.41	157.2 ± 23.2	nd.	nd.	nd.	nd.
RPN3	tr.	0.052 ± 0.009	0.788 ± 0.067	0.680 ± 0.069	18.19 ± 0.52	122.4 ± 11.9	nd.	nd.	nd.	nd.
RPN4	tr.	0.088 ± 0.013	0.768 ± 0.108	1.008 ± 0.129	14.73 ± 0.15	117.6 ± 11.1	nd.	nd.	nd.	nd.
RPN5	0.603 ± 0.024	0.040 ± 0.001	0.278 ± 0.003	0.141 ± 0.006	20.62 ± 4.55	71.7 ± 5.1	nd.	nd.	nd.	nd.
RPN6	1.968 ± 0.077	0.234 ± 0.021	1.216 ± 0.037	1.108 ± 0.050	10.11 ± 1.16	108.5 ± 14.7	nd.	nd.	nd.	nd.
RPN7	0.308 ± 0.026	0.019 ± 0.002	0.828 ± 0.039	0.432 ± 0.032	7.443 ± 0.639	120.0 ± 17.4	nd.	nd.	nd.	nd.
RPN8	tr.	0.011 ± 0.002	0.175 ± 0.021	0.256 ± 0.014	26.23 ± 1.08	84.7 ± 7.4	nd.	nd.	nd.	nd.
RPN9	0.363 ± 0.021	0.103 ± 0.005	0.558 ± 0.036	0.179 ± 0.017	3.630 ± 0.059	85.5 ± 6.3	nd.	nd.	nd.	nd.
RPN10	tr.	tr.	0.131 ± 0.010	0.644 ± 0.058	13.37 ± 1.95	102.2 ± 2.7	nd.	nd.	nd.	nd.

Table S4 (*continued*)

Sample	D3	O1	P1	P2	P3	P4	P5	P6	P7	P8
DQP1	0.455 ± 0.211	1.542 ± 0.062	32.71 ± 3.64	13.89 ± 0.59	9.34 ± 1.38	4.884 ± 0.340	2.388 ± 0.123	11.39 ± 1.11	8.92 ± 0.94	1.342 ± 0.245
DQP2	3.020 ± 0.104	0.899 ± 0.063	25.68 ± 1.40	10.45 ± 0.22	10.45 ± 0.94	6.088 ± 0.716	1.944 ± 0.170	10.09 ± 1.40	6.176 ± 1.342	0.876 ± 0.122
DQP3	1.217 ± 0.013	1.013 ± 0.084	26.72 ± 1.66	9.79 ± 0.01	15.06 ± 0.32	7.896 ± 0.300	2.116 ± 0.151	12.258 ± 1.158	5.606 ± 0.374	2.142 ± 0.086
DQP4	1.426 ± 0.242	1.366 ± 0.036	29.17 ± 1.43	14.76 ± 0.67	4.970 ± 0.286	2.378 ± 0.186	2.055 ± 0.235	8.06 ± 1.71	7.625 ± 1.668	0.935 ± 0.005
DQP5	1.712 ± 0.046	0.298 ± 0.012	21.48 ± 2.48	10.67 ± 0.03	6.488 ± 0.896	3.553 ± 0.492	1.576 ± 0.108	6.878 ± 0.535	4.713 ± 0.574	0.758 ± 0.054
DQP6	1.629 ± 0.057	0.746 ± 0.031	29.20 ± 0.88	8.83 ± 0.24	8.80 ± 1.55	4.524 ± 0.688	2.142 ± 0.153	11.92 ± 1.58	10.13 ± 1.36	1.339 ± 0.144
DQP7	1.316 ± 0.114	1.165 ± 0.067	18.76 ± 1.63	6.971 ± 0.998	8.57 ± 0.60	4.664 ± 0.253	1.441 ± 0.133	7.955 ± 0.893	4.602 ± 0.484	1.047 ± 0.082
DQP8	1.307 ± 0.113	1.006 ± 0.022	24.34 ± 1.29	9.51 ± 0.75	11.51 ± 0.50	6.302 ± 0.263	1.879 ± 0.331	10.23 ± 1.67	5.526 ± 0.444	1.373 ± 0.072
DQP9	1.420 ± 0.101	1.234 ± 0.037	22.69 ± 0.58	7.939 ± 0.434	9.44 ± 1.26	5.117 ± 0.222	1.722 ± 0.389	9.56 ± 0.75	6.219 ± 0.755	1.178 ± 0.086
DQP10	1.534 ± 0.154	1.179 ± 0.157	22.67 ± 0.58	11.66 ± 0.66	10.03 ± 0.23	5.667 ± 0.166	1.735 ± 0.044	8.05 ± 0.64	3.560 ± 0.729	1.022 ± 0.009
DQP11	1.068 ± 0.008	1.054 ± 0.140	26.26 ± 2.62	11.95 ± 0.99	8.11 ± 0.33	4.157 ± 0.470	1.931 ± 0.260	8.96 ± 2.35	6.412 ± 1.524	1.249 ± 0.085
DQP12	3.008 ± 0.347	0.819 ± 0.102	19.43 ± 1.41	9.58 ± 0.51	8.29 ± 0.96	4.555 ± 0.289	1.479 ± 0.138	6.989 ± 0.769	3.435 ± 0.245	0.969 ± 0.120
DQP13	1.778 ± 0.039	1.266 ± 0.086	19.57 ± 0.97	6.558 ± 0.458	6.308 ± 0.474	3.289 ± 0.171	1.445 ± 0.121	7.827 ± 0.731	6.224 ± 1.113	0.916 ± 0.146
DQP14	1.913 ± 0.033	1.570 ± 0.135	18.66 ± 0.02	7.155 ± 0.042	12.90 ± 0.17	6.724 ± 0.217	1.530 ± 0.005	9.14 ± 0.04	2.835 ± 0.076	1.880 ± 0.102
DQP15	1.977 ± 0.122	1.426 ± 0.039	18.83 ± 0.88	8.09 ± 0.50	5.652 ± 0.271	3.020 ± 0.053	1.381 ± 0.064	6.593 ± 0.254	4.973 ± 0.150	0.748 ± 0.127
DQP16	1.365 ± 0.119	1.276 ± 0.317	20.21 ± 1.18	10.07 ± 0.94	8.76 ± 0.79	4.754 ± 0.434	1.542 ± 0.096	7.264 ± 0.350	3.453 ± 0.132	1.085 ± 0.095
DQP17	1.306 ± 0.013	0.753 ± 0.021	27.68 ± 1.02	9.25 ± 0.61	6.912 ± 0.384	3.663 ± 0.270	1.999 ± 0.188	10.47 ± 0.67	9.55 ± 0.06	0.945 ± 0.119
DQP18	1.613 ± 0.022	0.719 ± 0.127	26.72 ± 1.66	9.79 ± 0.01	15.06 ± 0.32	7.896 ± 0.300	2.116 ± 0.251	12.26 ± 1.76	5.606 ± 1.274	2.142 ± 0.086
DQP19	1.430 ± 0.151	0.768 ± 0.056	21.81 ± 1.24	10.00 ± 0.14	13.82 ± 0.40	7.365 ± 0.180	1.761 ± 0.091	9.56 ± 0.62	2.701 ± 0.572	1.851 ± 0.084
DQP20	1.145 ± 0.102	1.557 ± 0.058	32.15 ± 2.69	13.90 ± 0.08	12.60 ± 1.11	7.182 ± 0.740	2.423 ± 0.155	12.11 ± 0.92	7.361 ± 0.818	1.221 ± 0.002
DQP21	1.509 ± 0.048	0.453 ± 0.022	24.04 ± 0.62	9.31 ± 0.73	10.10 ± 0.76	5.244 ± 0.294	1.827 ± 0.113	9.76 ± 0.02	5.970 ± 0.341	1.490 ± 0.247
DQP22	1.614 ± 0.088	0.269 ± 0.025	17.96 ± 0.96	6.881 ± 0.398	7.519 ± 2.098	4.238 ± 0.216	1.365 ± 0.111	7.316 ± 1.073	4.519 ± 0.307	0.774 ± 0.182
DQP23	4.074 ± 0.719	0.793 ± 0.097	19.85 ± 1.28	9.11 ± 0.96	9.60 ± 0.78	5.418 ± 0.338	1.536 ± 0.191	7.786 ± 1.139	3.535 ± 0.220	0.980 ± 0.146
DQP24	1.623 ± 0.130	0.724 ± 0.058	23.66 ± 0.54	12.80 ± 1.21	9.63 ± 0.57	5.173 ± 0.238	1.791 ± 0.157	7.854 ± 0.433	3.608 ± 0.279	1.245 ± 0.141
DQP25	1.052 ± 0.028	0.773 ± 0.076	27.27 ± 0.22	10.33 ± 0.04	13.76 ± 0.17	7.248 ± 0.177	2.124 ± 0.011	11.87 ± 0.03	6.084 ± 0.177	1.925 ± 0.062
DQP26	1.064 ± 0.059	1.518 ± 0.037	32.53 ± 0.36	13.71 ± 0.59	9.53 ± 0.36	5.105 ± 0.372	2.380 ± 0.121	11.44 ± 0.82	8.851 ± 0.732	1.248 ± 0.132
DQP27	2.186 ± 0.096	0.944 ± 0.021	20.83 ± 2.11	10.50 ± 0.81	8.12 ± 1.17	4.737 ± 0.126	1.569 ± 0.115	7.154 ± 0.238	3.807 ± 0.182	0.675 ± 0.043
DQP28	1.821 ± 0.066	1.274 ± 0.092	22.51 ± 1.03	10.72 ± 0.10	11.32 ± 1.14	6.174 ± 0.313	1.752 ± 0.183	8.79 ± 0.53	3.605 ± 0.483	1.373 ± 0.116
DQP29	1.064 ± 0.061	1.336 ± 0.025	21.00 ± 2.48	11.20 ± 0.26	8.37 ± 0.42	4.451 ± 0.358	1.586 ± 0.219	6.994 ± 0.741	3.373 ± 0.569	1.130 ± 0.257
DQP30	1.374 ± 0.038	0.538 ± 0.035	25.18 ± 1.03	7.778 ± 0.219	7.034 ± 0.632	4.026 ± 0.404	1.835 ± 0.221	10.04 ± 1.38	8.83 ± 2.93	0.664 ± 0.017
RSM1	4.376 ± 0.798	0.145 ± 0.007	0.491 ± 0.023	nd.	nd.	0.153 ± 0.024	0.643 ± 0.077	3.246 ± 0.236	6.868 ± 0.731	nd.
RSM2	7.095 ± 0.032	0.261 ± 0.011	0.254 ± 0.022	nd.	nd.	4.505 ± 0.624	2.897 ± 0.256	2.050 ± 0.374	41.52 ± 1.37	nd.
RSM3	10.94 ± 0.92	0.149 ± 0.010	0.387 ± 0.060	nd.	nd.	6.718 ± 0.440	3.207 ± 0.070	2.399 ± 0.298	51.60 ± 5.91	nd.
RSM4	4.273 ± 0.553	0.346 ± 0.010	0.252 ± 0.023	nd.	nd.	2.616 ± 0.113	0.878 ± 0.118	1.886 ± 0.070	23.47 ± 2.43	nd.
RSM5	9.49 ± 1.24	0.120 ± 0.010	0.260 ± 0.027	nd.	nd.	3.482 ± 0.243	2.481 ± 0.047	3.163 ± 0.589	42.68 ± 3.51	nd.
RSM6	7.566 ± 0.638	0.278 ± 0.014	0.350 ± 0.045	nd.	nd.	2.446 ± 0.283	1.478 ± 0.190	2.501 ± 0.214	38.68 ± 5.81	nd.
RSM7	4.116 ± 0.114	0.257 ± 0.012	0.451 ± 0.067	nd.	nd.	5.533 ± 0.544	3.434 ± 0.071	2.023 ± 0.152	40.35 ± 4.33	nd.
RSM8	6.899 ± 0.790	0.291 ± 0.038	0.309 ± 0.018	tr.	nd.	1.838 ± 0.269	1.439 ± 0.108	1.740 ± 0.160	31.39 ± 2.25	nd.
RSM9	9.92 ± 0.85	0.375 ± 0.053	0.335 ± 0.009	nd.	nd.	4.308 ± 0.462	3.293 ± 0.506	2.646 ± 0.313	37.67 ± 2.77	nd.
RSM10	8.55 ± 1.43	0.923 ± 0.064	0.436 ± 0.033	tr.	nd.	3.521 ± 0.280	1.564 ± 0.180	2.011 ± 0.236	36.32 ± 4.02	nd.
RPN1	nd.	nd.	nd.	nd.	nd.	nd.	nd.	nd.	nd.	nd.
RPN2	nd.	nd.	nd.	nd.	nd.	nd.	nd.	nd.	nd.	nd.
RPN3	nd.	nd.	nd.	nd.	nd.	nd.	nd.	nd.	nd.	nd.
RPN4	nd.	nd.	nd.	nd.	nd.	nd.	nd.	nd.	nd.	nd.
RPN5	nd.	nd.	nd.	nd.	nd.	nd.	nd.	nd.	nd.	nd.
RPN6	nd.	nd.	nd.	nd.	nd.	nd.	nd.	nd.	nd.	nd.
RPN7	nd.	nd.	nd.	nd.	nd.	nd.	nd.	nd.	nd.	nd.
RPN8	nd.	nd.	nd.	nd.	nd.	nd.	nd.	nd.	nd.	nd.
RPN9	nd.	nd.	nd.	nd.	nd.	nd.	nd.	nd.	nd.	nd.
RPN10	nd.	nd.	nd.	nd.	nd.	nd.	nd.	nd.	nd.	nd.

Table S4 (*continued*)

Sample	S1	S2	S3	S4	S5	S6	S7	Total content (%)
DQP1	7.283 ± 0.176	38.58 ± 0.49	3.461 ± 0.082	28.36 ± 2.81	0.975 ± 0.048	1.767 ± 0.078	5.352 ± 0.737	57.0
DQP2	4.828 ± 0.009	32.70 ± 0.16	2.975 ± 0.213	23.16 ± 1.67	0.954 ± 0.066	1.688 ± 0.025	5.624 ± 0.698	56.1
DQP3	6.852 ± 0.250	34.85 ± 0.46	2.441 ± 0.168	16.34 ± 2.05	0.924 ± 0.094	1.756 ± 0.167	5.520 ± 0.077	54.7
DQP4	8.93 ± 0.28	39.70 ± 2.22	2.656 ± 0.230	26.07 ± 1.83	0.956 ± 0.037	2.143 ± 0.323	6.032 ± 0.121	69.9
DQP5	6.299 ± 0.504	37.71 ± 1.45	3.060 ± 0.245	21.88 ± 0.78	0.638 ± 0.038	2.321 ± 0.147	4.052 ± 0.368	57.4
DQP6	6.303 ± 0.352	26.82 ± 0.09	2.357 ± 0.331	22.48 ± 1.37	0.584 ± 0.057	1.592 ± 0.154	4.308 ± 0.222	63.6
DQP7	7.128 ± 0.308	31.20 ± 0.18	2.510 ± 0.218	21.39 ± 1.12	0.614 ± 0.043	1.578 ± 0.056	4.937 ± 0.280	52.9
DQP8	5.870 ± 0.293	34.24 ± 0.67	2.825 ± 0.042	33.44 ± 2.76	0.772 ± 0.060	2.021 ± 0.086	7.142 ± 0.989	55.0
DQP9	5.470 ± 0.039	36.55 ± 0.75	3.220 ± 0.116	23.70 ± 1.01	1.055 ± 0.139	1.781 ± 0.123	5.583 ± 0.279	61.0
DQP10	7.276 ± 0.277	36.46 ± 1.22	2.881 ± 0.216	21.68 ± 1.09	0.700 ± 0.001	2.148 ± 0.201	6.098 ± 0.174	55.3
DQP11	7.778 ± 0.038	33.96 ± 0.43	1.998 ± 0.223	22.70 ± 0.03	0.581 ± 0.032	2.057 ± 0.191	4.148 ± 0.069	58.7
DQP12	6.120 ± 0.390	34.26 ± 0.45	2.732 ± 0.105	27.13 ± 1.68	0.844 ± 0.054	2.020 ± 0.362	5.416 ± 0.583	63.1
DQP13	5.428 ± 0.414	33.68 ± 1.88	3.035 ± 0.065	18.46 ± 1.11	0.816 ± 0.136	1.532 ± 0.009	5.250 ± 0.752	54.9
DQP14	6.402 ± 0.903	40.39 ± 4.23	3.531 ± 0.485	37.32 ± 1.97	1.272 ± 0.142	2.616 ± 0.294	6.608 ± 1.403	55.2
DQP15	6.399 ± 0.324	35.62 ± 0.31	2.606 ± 0.151	19.80 ± 0.79	0.843 ± 0.023	2.244 ± 0.045	4.018 ± 0.212	57.1
DQP16	6.795 ± 0.010	36.98 ± 3.02	2.478 ± 0.230	29.39 ± 2.73	0.788 ± 0.074	2.071 ± 0.071	6.593 ± 0.079	63.5
DQP17	7.230 ± 0.290	31.35 ± 1.88	2.535 ± 0.088	22.77 ± 3.12	0.673 ± 0.058	2.165 ± 0.045	5.676 ± 0.285	60.1
DQP18	4.223 ± 0.399	24.71 ± 0.01	2.065 ± 0.043	15.17 ± 0.06	0.662 ± 0.045	1.450 ± 0.234	5.245 ± 0.674	58.1
DQP19	7.881 ± 0.140	36.20 ± 0.14	2.430 ± 0.299	22.74 ± 2.19	0.553 ± 0.035	2.127 ± 0.088	4.371 ± 0.075	69.5
DQP20	8.16 ± 0.03	38.13 ± 1.77	3.016 ± 0.131	24.33 ± 3.33	0.648 ± 0.094	2.547 ± 0.106	6.314 ± 0.344	66.6
DQP21	7.783 ± 0.007	31.63 ± 0.05	2.270 ± 0.205	27.51 ± 2.00	0.523 ± 0.121	1.289 ± 0.025	4.004 ± 0.677	53.0
DQP22	7.057 ± 0.405	35.54 ± 5.81	2.407 ± 0.262	19.01 ± 1.28	0.585 ± 0.003	1.567 ± 0.270	5.209 ± 0.630	57.8
DQP23	6.305 ± 0.263	31.01 ± 2.52	3.511 ± 0.224	20.86 ± 1.88	0.735 ± 0.058	2.097 ± 0.128	5.154 ± 0.724	54.7
DQP24	5.768 ± 0.434	34.44 ± 2.27	3.391 ± 0.064	23.26 ± 1.83	0.659 ± 0.016	2.083 ± 0.190	4.807 ± 0.380	52.9
DQP25	5.274 ± 0.709	30.21 ± 0.98	2.226 ± 0.176	19.90 ± 1.47	0.514 ± 0.086	2.487 ± 0.191	4.091 ± 0.214	63.4
DQP26	5.452 ± 0.182	31.14 ± 1.95	3.386 ± 0.040	29.44 ± 1.10	1.242 ± 0.090	1.671 ± 0.063	5.793 ± 0.427	59.1
DQP27	5.194 ± 0.328	36.05 ± 0.12	2.845 ± 0.246	20.35 ± 0.94	0.771 ± 0.061	2.789 ± 0.220	4.883 ± 0.384	53.9
DQP28	5.437 ± 0.048	32.49 ± 1.14	2.342 ± 0.027	22.30 ± 0.05	0.615 ± 0.038	2.565 ± 0.088	5.034 ± 0.392	52.6
DQP29	7.134 ± 0.057	29.11 ± 0.50	1.731 ± 0.065	25.76 ± 0.80	0.534 ± 0.054	1.720 ± 0.226	4.244 ± 0.278	56.2
DQP30	4.196 ± 0.015	27.48 ± 0.46	2.522 ± 0.084	15.16 ± 0.15	0.591 ± 0.069	1.576 ± 0.026	4.155 ± 0.071	58.0
RSM1	nd.	nd.	nd.	nd.	nd.	nd.	nd.	21.4
RSM2	nd.	nd.	nd.	nd.	nd.	nd.	nd.	32.8
RSM3	nd.	nd.	nd.	nd.	nd.	nd.	nd.	27.3
RSM4	nd.	nd.	nd.	nd.	nd.	nd.	nd.	33.9
RSM5	nd.	nd.	nd.	nd.	nd.	nd.	nd.	31.2
RSM6	nd.	nd.	nd.	nd.	nd.	nd.	nd.	31.8
RSM7	nd.	nd.	nd.	nd.	nd.	nd.	nd.	31.6
RSM8	nd.	nd.	nd.	nd.	nd.	nd.	nd.	23.1
RSM9	nd.	nd.	nd.	nd.	nd.	nd.	nd.	27.3
RSM10	nd.	nd.	nd.	nd.	nd.	nd.	nd.	29.1
RPN1	9.88 ± 1.04	31.95 ± 4.02	3.320 ± 0.126	39.05 ± 1.56	0.612 ± 0.052	1.117 ± 0.068	5.958 ± 0.758	24.6
RPN2	14.82 ± 1.77	38.42 ± 2.73	6.546 ± 0.169	45.47 ± 6.77	1.000 ± 0.137	1.129 ± 0.089	15.56 ± 0.41	30.3
RPN3	19.60 ± 2.18	39.98 ± 2.07	8.898 ± 1.015	48.61 ± 2.35	1.270 ± 0.101	0.612 ± 0.031	15.29 ± 1.19	27.7
RPN4	24.66 ± 2.97	41.75 ± 6.18	8.781 ± 0.211	42.73 ± 4.88	0.984 ± 0.135	1.439 ± 0.071	12.41 ± 1.58	26.7
RPN5	14.50 ± 1.76	41.36 ± 5.14	9.486 ± 1.388	45.08 ± 4.84	1.054 ± 0.076	nd.	14.03 ± 1.18	21.9
RPN6	20.58 ± 2.96	44.69 ± 6.21	9.486 ± 0.923	36.26 ± 4.66	1.070 ± 0.144	0.521 ± 0.031	10.86 ± 1.40	24.7
RPN7	17.21 ± 0.86	53.70 ± 7.65	11.799 ± 2.633	47.04 ± 3.55	1.262 ± 0.058	1.274 ± 0.030	17.89 ± 0.71	28.0
RPN8	18.15 ± 1.47	45.86 ± 2.81	8.467 ± 0.217	48.22 ± 4.34	1.168 ± 0.120	1.799 ± 0.244	15.52 ± 2.05	25.1
RPN9	13.64 ± 0.99	34.10 ± 3.68	6.272 ± 0.103	40.77 ± 5.41	0.835 ± 0.087	0.988 ± 0.108	12.86 ± 0.91	20.0
RPN10	18.66 ± 1.99	39.79 ± 4.44	5.527 ± 0.322	32.30 ± 4.08	0.941 ± 0.058	0.870 ± 0.100	12.56 ± 1.26	22.7

[a] The analyte abbreviations as given in **Table 1**.
[b] Trace.
[c] Not detected.
